# A molecular dynamics-based algorithm for evaluating the glycosaminoglycan mimicking potential of synthetic, homogenous, sulfated small molecules

**DOI:** 10.1371/journal.pone.0171619

**Published:** 2017-02-09

**Authors:** Balaji Nagarajan, Nehru Viji Sankaranarayanan, Bhaumik B. Patel, Umesh R. Desai

**Affiliations:** 1 Institute for Structural Biology, Drug Discovery and Development and Department of Medicinal Chemistry, Virginia Commonwealth University, Richmond, Virginia, United States of America; 2 Hunter Holmes Muire VA Medical Center, Richmond, Virginia, United States of America; 3 Division of Hematology, Oncology, and Palliative Care, Department of Internal Medicine and Massey Cancer Center, Virginia Commonwealth University, Richmond, Virginia, United States of America; University of Patras, GREECE

## Abstract

Glycosaminoglycans (GAGs) are key natural biopolymers that exhibit a range of biological functions including growth and differentiation. Despite this multiplicity of function, natural GAG sequences have not yielded drugs because of problems of heterogeneity and synthesis. Recently, several homogenous non-saccharide glycosaminoglycan mimetics (NSGMs) have been reported as agents displaying major therapeutic promise. Yet, it remains unclear whether sulfated NSGMs structurally mimic sulfated GAGs. To address this, we developed a three-step molecular dynamics (MD)-based algorithm to compare sulfated NSGMs with GAGs. In the first step of this algorithm, parameters related to the range of conformations sampled by the two highly sulfated molecules as free entities in water were compared. The second step compared identity of binding site geometries and the final step evaluated comparable dynamics and interactions in the protein-bound state. Using a test case of interactions with fibroblast growth factor-related proteins, we show that this three-step algorithm effectively predicts the GAG structure mimicking property of NSGMs. Specifically, we show that two unique dimeric NSGMs mimic hexameric GAG sequences in the protein-bound state. In contrast, closely related monomeric and trimeric NSGMs do not mimic GAG in either the free or bound states. These results correspond well with the functional properties of NSGMs. The results show for the first time that appropriately designed sulfated NSGMs can be good structural mimetics of GAGs and the incorporation of a MD-based strategy at the NSGM library screening stage can identify promising mimetics of targeted GAG sequences.

## Introduction

Glycosaminoglycans (GAGs), major constituents of the extracellular matrix, participate in regulating many different physiological and pathological processes by targeting a broad spectrum of proteins [1,2]. These negatively charged polymers recognize target proteins on the basis of number, density and distribution of sulfate groups. Because the biosynthesis of GAGs is a template-less process, nature tends to produce a large number of sulfation patterns in GAGs. Further, conformational biases of individual saccharide residues, e.g., ^1^C_4_ and ^2^S_O_ of iduronic acid, introduce additional diversity to the GAG scaffold. This implies that a GAG sequence as small as a hexasaccharide can exist in thousands of possible distinct topologies [3] Although not all of these sequences are likely to induce a biological function, the massive heterogeneity present in a typical GAG population appears to be important for ensuring a higher probability of functional success, e.g., cell growth and migration, morphogenesis, inflammation and immunity [2,4].

**Fig 1:**
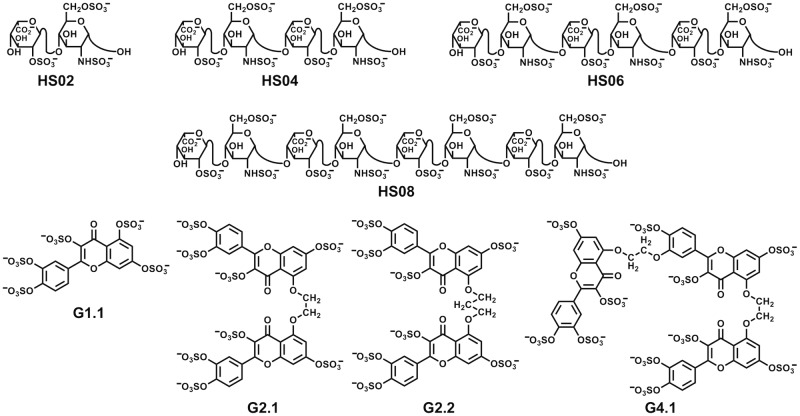
Structures of heparan sulfate (HS) sequences of varying lengths (HS02 to HS08) and non-saccharide glycosaminoglycan mimetics (NSGMs) G1.1, G2.1, G2.2 and G4.1. G1.1 can be considered as monomeric NSGM; G2.1 and G2.2 are approximately dimeric and G4.1 can be thought of as tetrameric NSGM.

A key feature of majority of GAG–protein systems is that the affinity and specificity of interaction relies primarily on the surface exposed sulfate groups and minimally on backbone atoms of GAGs. Some time ago we hypothesized that it may be more advantages to develop homogeneous, synthetic molecules containing appropriate number of sulfate groups as functional mimetics of GAGs because of the well-known difficulties with synthesizing or preparing/purifying GAG sequences [5]. This hypothesis then led to the synthesis of several homogeneous non-saccharide glycosaminoglycan mimetics (NSGMs, see Fig 1), which displayed major promise in modulating a number of biological processes [6–9]. The NSGMs studied so far have been found to engage their protein targets by means of hydrophobic, hydrogen bonding and Columbic forces, thereby affording considerable selectivity of recognition [5].

NSGMs prepared to date possess an aromatic backbone, whereas GAGs are based on the saccharide scaffold. NSGMs bear sulfate groups on phenolic groups, whereas GAGs bear sulfates on alcoholic or amine groups. NSGMs are generally achiral, whereas GAGs possess multiple chiral centers. These diametric differences bring forth a fundamental question on whether NSGMs are structurally equivalent or similar to GAGs. In fact, the number of NSGMs being studied in the literature is growing fast [6–12] and there is a strong possibility of developing drugs and/or chemical probes based on the promising NSGM leads identified to date. Further, the future of NSGMs as a technology is also promising because of their easier synthesis [5,7,9,11,13] and ability to apparently mimic GAG sequences at will. Thus, it is important to address the question on whether NSGMs structurally mimic GAGs effectively. Yet, tools for addressing this have not been established. In fact, availability of such tools may catapult the design of better NSGMs.

Recently, we studied a small library of NSGMs for inhibition of cancer stem cells (CSCs) [11]. Screening a library of 53 NSGMs from 12 different scaffolds resulted in identification of G2.1 and G2.2 (Fig 1) as inhibitors of CSCs. Interestingly, neither G1.1 nor G4.1 (Fig 1) were found to exhibit this activity suggesting a high level of selectivity. Why do G2.1 and G2.2 function as CSCs inhibitors but not G1.1 and G4.1? Is it possible that G2.1 and G2.2 mimic a GAG sequence of defined chain length, e.g., a heparan sulfate (HS) hexasaccharide (HS06)? This may be the case because HS sequences are known to regulate growth by binding to fibroblast growth factor 2 (FGF2) and/or its receptor (FGFR1) [14,15]?

A traditional approach to evaluate structural equivalence of GAGs is to compare similarity of sulfate groups in static structures [16,17]. However, sulfate moieties, especially present on GAGs, exhibit considerable dynamism, which is likely to be important in inducing functional properties. Thus, we utilized molecular dynamics (MD) to study the behavior of NSGMs (i.e., G1.4, G2.1, G2.2 and G4.1) and compared it with HS06 in free and protein-bound states This led to a three-step algorithm that helped predict GAG mimicking ability of different NSGMs. The algorithm predicted that although G2.1 and G2.2 have very similar structures, G2.2 mimics HS06 better than G2.1 in solution, while G1.1 and G4.1 exhibit a completely different profile. In the protein-bound form (FGF2 and FGF2–FGFR1 complex), G2.2 is predicted to interact with residues that engage HS06 and these complexes are better stabilized than equivalent ones with G2.1. These results provide a structural foundation for the literature report on the functional activity of NSGMs as anti-CSC agents. This is the first report on the development of a detailed computational algorithm for assessing equivalence of NSGMs with natural GAG sequences. We posit that this algorithm, or variants thereof, can now be implemented on a high-throughput scale for discovering/designing novel NSGMs.

## Methods

### Molecular dynamics (MD) of NSGMs

The initial models of NSGMs were built using SYBYLX2.1 (Tripos Associates, St. Louis, MO). Gasteiger-Hückel charges were assigned to the molecules and then each NSGM was minimized using conjugate gradient method for 10,000 iterations. Explicit MD simulations were then carried out using the AMBER14 package [18,19], using periodic boundary condition and long range interactions were calculated using particle Mesh Ewald method, which utilizes a generalized Amber force field (GAFF) for small organic molecules [20]. This force field has been optimized for a diverse range of organic scaffolds by employing a semi-empirical AM1-BCC charge model [21]. Initial models of NSGMs were loaded to the antechamber module of AMBER14 to assign appropriate charge and torsional angle parameters [22] and then neutralized by Na^+^ counter ions to give a net charge of zero. The solute molecules were set in center and were solvated in a cuboid periodic box of TIP3P water molecules [23] with a minimum distance of 10 Å between the wall and any atom of the solute. Initial parameters and co-ordinates files were generated followed by a two-step minimization process. In first step the solute and the Na^+^ ions were restrained using a harmonic potential of 100 kcal/(mol Å^2^). The water molecules were relaxed using 500 cycles of steepest descent and 1500 cycles of conjugate gradient method. In the second step the whole system was relaxed to 2500 cycles of conjugate gradient minimization. Following this the system was brought to constant temperature (300 K) using the Berendsen temperature coupling with time constant 2 ps and then brought to constant pressure (1 atm). Finally the system was equilibrated (at NPT) without any restraints. All these phases were performed for a total time period of 1 ns with 2 fs integration time step. The NPT MD simulation was carried out after equilibrating the system, with integration time step of 1 fs for a total time period of 20 ns, during which the ensemble coordinates were collected at every 1 ps. The covalent bonds involving hydrogen atoms were constrained using SHAKE algorithm throughout the simulation.

### MD of heparan sulfate hexasaccharide (HS06)

The exploration of HS06 dynamics in explicit water was performed using AMBER14 with GLYCAM06h force field parameters [24]. Two independent initial HS06 starting structures for this simulation were taken from the experimental NMR structure (PDBID: 1HPN) [25]. This structure has a repeating sequence of (IdoA2S-GlcNS6S)_3_ with IdoA2S existing in either chair ^1^C_4_ or skew boat ^2^S_O_ conformations, as shown in (Fig A in S1 File). The initial structures were loaded in Leap and the system was neutralized with appropriate number of Na^+^ counter ions. The molecules were solvated in a periodic box of TIP3P water molecules [23] with a distance of 10 Å between the box edge and solute surface. A protocol similar to that of NSGM simulations was followed here (see the section above), except for an additional weak torsional restrain to keep the pyranose ring of IdoA2S in either ^2^S_O_ or ^1^C_4_ conformation throughout the simulation [26,27].

### Molecular docking of NSGMs binding to proteins

GOLD v5.2 (from Cambridge Crystallographic Data Center, Cambridge, UK) was employed to study the interaction of NSGMs with proteins [28]. The three dimensional structures of FGF2 (PDBID: 1BFC) [29] and FGF2–FGFR1 (PDBID: 1FQ9) [30] complex were obtained from Protein Data Bank (http://www.rcsb.org). Protein preparation was carried out using SYBYL X2.1, which included addition of hydrogen and missing atoms, protonation of residues, removal of steric clashes and energy minimization. The centroid of the GAG-binding sites in these proteins (FGF2 and FGF2–FGFR1 complex) was taken as the center and 18 Å radius was defined as the binding site. Docking was performed for 300 genetic algorithm runs with 100,000 iterations and early termination option was disabled. The GOLD fitness score was calculated from the contributions of hydrogen bond and van der Waals interactions between the protein and ligand [28]. From the GOLD based docking, the best sampled pose (highest GOLD score) with <2.5 Å root mean square deviation (rmsd) were selected for further studies.

### MD of NSGM–protein complexes

The best docked structures of the FGF2 and FGF2–FGFR1 complexes with NSGM obtained following GOLD-based docking were prepared for MD using the Leap module of the AMBER14 suite [18]. The molecules was parameterized using the antechamber module, in which AM1-BCC was used to assign the atomic charges [22]. Force field parameters for the NSGMs were generated using GAFF [20]. The total charge of the complex was brought to zero by adding an appropriate number of the Na^+^ counter ions. AMBER-ff99SB force field [31] was utilized for the protein receptors (FGF2 and FGF2–FGFR1 complex). The charge-neutralized NSGM–protein complex was placed in the center of a TIP3P cuboid water box [23] with minimum distance of 12 Å between the box wall to any atom of the comple. MD simulations were performed using AMBER14 and periodic boundary conditions were applied to avoid the edge effects. Particle Mesh Ewald module was employed for calculating long-range interactions. The covalent bonds involving hydrogen atoms were constrained using SHAKE algorithm. The NSGM–protein complexes were energy minimized in two steps with the non-bonded cut off 10 Å to remove steric hindrance. In the first step, the solute atoms including the Na^+^ counter ions were restrained using a harmonic potential with the force constant of 100 kcal/(mol Å^2^). The water molecules were relaxed using 500 cycles of steepest descent and 2000 cycles of conjugate gradient method. In the second step, the whole system was relaxed to conjugate gradient minimization of 2500 cycles without any restrain.

For MD simulations, the system was equilibrated in three phases including i) raising temperature to 300 K using the Berendsen temperature coupling with time constant 2 ps, ii) equilibrating pressure to 1 atm, and iii) equilibrating all atoms at the NPT without any restrains. All of these phases were performed with in 1 ns. Following this equilibration, a production run of 20 ns was initiated in explicit solvent environment using NPT ensemble with the integration time step of 1 fs. The trajectory files were collected at every 1 ps for further analysis by ambertools14 [32] and VMD software [33]. Clustering analysis was carried out using MMTSB [34].

### HS06-protein complex docking

To study the interaction of HS06 with FGF2 and FGF2–FGFR1 complex, we utilized a computational protocol developed earlier called combinatorial virtual library screening (CVLS) strategy [35]. Protein molecules prepared for docking NSGMs were used for this simulation. For each protein target, a 300 genetic algorithm run was employed in triplicate and the best 6 docked poses were scored using GOLD score, a measure of strength of interaction between the two. Co-ordinates for HS06 containing IdoA2S in both ^1^C_4_ and ^2^S_O_ conformations were first generated followed by simulation of interactions using Discovery studio visualizer (Accelrys Software, Cambridge, England).

### MD of HS06-protein complexes

The best docked structures of the FGF2 and FGF2–FGFR1 complexes with HS06 (IdoA2S-GlcNS6S)_3_ sequences were prepared for MD using the Leap module of the AMBER14 suite. The ff99SB force field was used for the parameterization of protein and HS06 force field parameters were created based on GLYCAM06h [24]. The simulation protocols were similar to those used for NSGMs complexes (see section above). In addition weak torsional restraints were applied to keep the pyranose ring of IdoA2S in the preferred puckering conformation in HS06.

### MM/PB(GB)SA binding free energy calculations

Binding free energy calculation of each NSGM–protein complex and HS06-protein complex was computed using the MM/PB(GB)SA method [36]. The ensembles of conformers from 10 ns to 20 ns in equal interval of time (10 ps) of total 1000 conformers were selected for the MMPBSA calculations. MMGBSA method employing single residue energy decomposition (SRED) was used to estimate the free energy contributions of the each residue contributing to binding. Both the energy calculations were performed with the default parameter settings by employing the Python version of MM/PB(GB)SA module from ambertools14 [32,36].

## Results and discussion

### Rationale for MD-based algorithm to assess GAG mimicking potential of NSGMs

Many reports support the concept that sulfated NSGMs appear to be functionally mimicking GAGs [6,8,11]. Nearly each of these studies has propagated this concept on the observation that NSGMs target the same binding sites on proteins as those targeted by GAGs. For example, the heparin-binding site of thrombin, factor XIIIa, factor XIa and plasmin is targeted by distinct sulfated NSGMs [6,13,37–39] Likewise, the heparin-binding site of antithrombin is the site of binding of another group of sulfated NSGMs [40,41]. Yet, whether NSGMs’ functional mimicking property arises from their close structural resemblance to GAGs has remained an open question.

Traditionally, NSGMs have been discovered either through functional screening or molecular modeling-based approaches [40,41]. In the latter category, design strategies have relied on assessing three-dimensional surface equivalence of sulfate groups on NSGMs and GAGs *in vacuo* [16,40,41]. NSGMs that display good overlap of sulfate groups in the static state with those of GAGs were deemed interesting and pursued for functional screening. Whereas such simple overlaps of one or more sulfate groups is useful for first approximations, true structural equivalence implies equivalence of static as well as dynamic states. More importantly, true mimicking properties can only arise if dynamic equivalence is present in the protein-bound state. Thus, we reasoned that a MD-based algorithm should be developed for assessing GAG mimicking potential of sulfated NSGMs. This algorithm would compare dynamic ensembles of the target NSGMs and GAG in water in both the free form as well as the protein-bound form.

Unfortunately, MD of highly sulfated molecules, e.g., GAGs and NSGMs, is challenging. In fact, very few studies have been performed involving MD of GAGs [42–46] and no studies have been undertaken for NSGMs. Key reasons for this include the difficulty of parameterization of sulfate group atoms, the significant flexibility around glycosidic linkage and conformational puckering of the pyranose rings [47,48]. Apart from this, a number of stereo-electronic effects also arise adding to the complexity of dynamical calculations [49]. Finally, it has been challenging to build a reliable force field that simulates interactions of all possible disaccharide pairs. Recently, GLYCAM06 force field has been implemented to resolve these challenges and aid application of MD to GAGs [24]. Here we exploit this recent advance in assessing GAG mimicking property of NSGMs.

### Rationale behind the selection of discrete group of NSGMs for comparison with GAGs

As discussed in the introductory section, from a library of 53 NSGMs, only two NSGMs containing the flavonoid scaffold, i.e., G2.1 and G2.2 (Fig 1), were found to selectively inhibit CSCs [11]. In contrast, G1.1 and G4.1 did not inhibit CSCs even at very high concentrations. Structurally, the four NSGMs are closely related to each other. G1.1 can be thought of as a monomer, G2.1 and G2.2 resemble a dimeric structure, and G4.1 is equivalent to a trimer. Between the two dimeric entities, G2.2 displayed higher potency than G2.1 suggesting a sensitive structural dependence of function.

The rationale behind screening NSGMs for modulation of CSCs was that GAGs are known to interact with several factors involved in growth and/or differentiation signaling [50–54] including fibroblast growth factor 2 (FGF2) and its receptor (FGFR1). These signaling proteins play critical roles in deciding the fate of a stem cell. Both interact with HS, a known regulator of stem cell growth. Although the exact mechanism of how HS modulates stem cell growth remains unclear, it is known to facilitate ternary complexation of FGF2 and FGFR1, thereby affecting growth and/or differentiation. In fact, HS06 was recently found to modulate CSCs with nearly equal potency as G2.1 and G2.2 [55]. Thus, soluble NSGMs, if mimicking HS, were expected to interfere with HS signaling, thereby inducing inhibition. This simplistic reasoning was supported by the discovery that G2.2 (and to a slightly lower extent G2.1) potently and selectively inhibited CSCs. However, whether G2.2 and G2.1 optimally mimic HS remained unestablished. Likewise, it is a bit counterintuitive that G4.1, which can be thought of as an overlapped combination of two G2.1 units, was found to be essentially inactive.

To understand this structure–activity relationship, we hypothesized that G2.2 and G2.1, but not G1.1 and G4.1, mimic HS06, which is already known to optimally engage FGF2–FGFR1 complex [30,56]. Implicit in this hypothesis is the idea that despite containing the dimeric G2.1-like structure, the trimeric G4.1 scaffold displays some feature that prevents recognition of the G2.1 structure embedded within it.

### MD reveals considerable conformational sampling by NSGMs and GAGs

To evaluate GAG mimicking property of the four structurally related NSGMs (Fig 1), we first performed all-atom MD of each agent in explicit water. Over a 20 ns MD run, 20000 conformations were collected so as to enable a comprehensive profile of the dynamic reorganization sampled by each NSGM. These conformations were clustered by K-means clustering method to group all conformations with RMSD of less than 2 Å [34]. The four NSGMs displayed a wide range of clusters suggesting widely different conformational dynamism (Fig B in S1 File). Whereas G1.1 and G4.1 were the least and most flexible of the four NSGMs, respectively, G2.1 and G2.2 displayed intermediate, but distinctly different, flexibility. *A priori* this flexibility appeared to arise from the various torsions sampled by the linker(s) between flavonoid moieties as well as combinatorial orientations sampled by the sulfate atoms. This implied that conformational dynamism of NSGMs, which is typically never factored into static interaction study (e.g., sulfate group overlap), is likely to be a major contributor to affinity, selectivity and function of NSGMs. Moreover in G2.2 the lowest energy structure and the centroid from maximum cluster are structurally similar as evidenced by an RMSD of only 2.40 Å (Fig C in S1 File), which is lower than that for other NSGMs.

**Fig 2:**
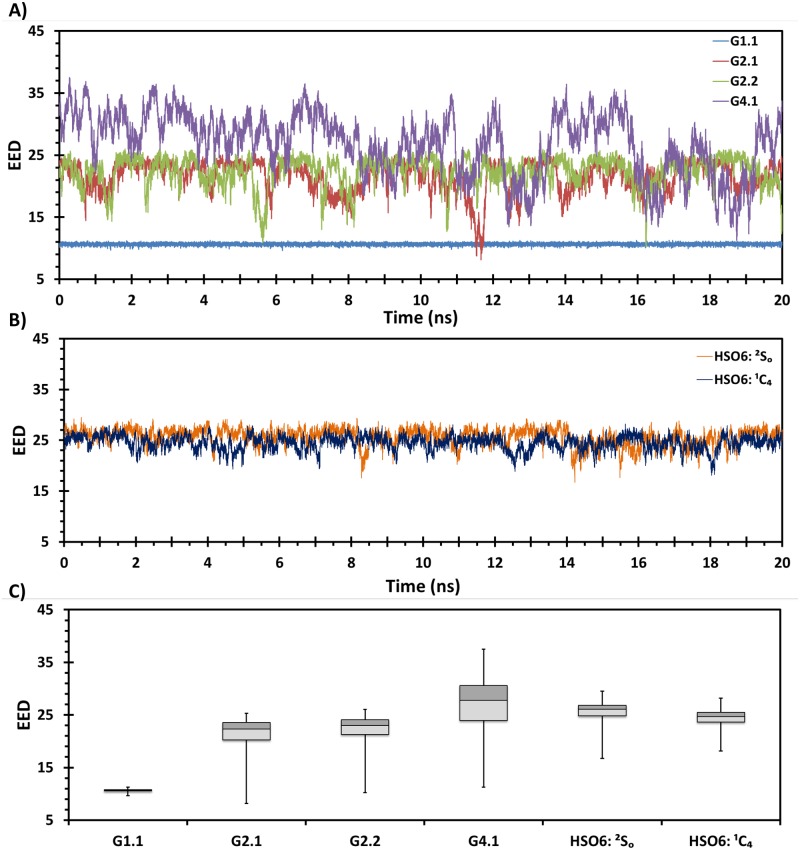
Molecular dynamics (MD)-based structural equivalence of NSGMs to HS06 in free solution using end-to-end distance (EED) as one of the comparable parameters. A) shows EED of each MD frame for all four NSGMs; B) shows EED for HS06 sequence containing IdoA2S in ^2^S_O_ and ^1^C_4_ conformations; and C) compiles the results across the MD simulation in a box plot.

The considerable conformational sampling induces a change in shape of the molecule, which can be partially captured by measuring the end-to-end distance (EED) for each NSGM (Fig D in S1 File). G2.1 and G2.2 always tended to exhibit extended conformations with an EED of ~25 Å shown in Fig 2A and Fig E in S1 File. In contrast, G4.1 displayed a tendency to fold into a globular structure (Fig 2A), which alludes to the possibility of hydrophobic interaction between aromatic rings. Finally, monomer G1.1 exhibited minimal change in shape (Fig 2A), as expected on the basis of its least number of flexible bonds. Apart from the EED, the conformational entropy of these four NSGMs was analyzed using principal component analysis (Fig F in S1 File) [57]. PCA is used to reduce the multidimensional coordinate space to a set of orthogonal vectors to clarify key variance of the coordinate space. We used PTRAJ/CPPTRAJ program to perform this analysis [57]. The collected ensemble of NSGMs coordinate space was subjected to covariance matrix calculations followed by diagonalization and projections to obtain the first three PCAs. This analysis indicated that the conformational diversity of G2.1 and G2.2 are almost similar and equally distributed in the coordinate phase space. G4.1 has the highest conformational entropy among the four due increased degrees of freedom associated with the additional two linkers.

Overall, the NSGMs G2.1 and G2.2 exhibited considerable similarity in conformational entropy of possessing extended and near extended conformers. In contrast, monomer G1.1 was essentially invariant, while G4.1 possessed much greater conformational entropy, despite having a similar core. These differences can be expected to induce differences in function, e.g., inhibition of CSCs.

### G2.1 and G2.2 in solution are similar to HS06

The above conformational analysis showed that G2.1 and G2.2 always tend to be in more extended form. Owing to additional degrees of freedom G4.1 tended to have mixed conformers. We were further interested in comparing these EEDs to those of the natural GAG heparan sulfate (Fig D in S1 File). Hexasaccharide HS06 (IdoA2S-GlcN2S6S)_3_ containing the most common sequence present in polymeric heparin possessed a distance of ~26 Å based on its NMR structure (PDBID: 1HPN), in which the IdoA2S residue displays both chair and boat forms. We studied the EED fluctuations of HS06 in explicit water and found a much more consistent overall 3D conformation (Fig 2B). Comparison of the dynamics over 20 ns shows that G2.1 and G2.2 tend to be in line with the HS06 dynamics, irrespective of whether IdoA2S is in the chair or boat form (Fig 2C). In contrast, G4.1 displays much more dramatic oscillations (Fig 2C).

To further assess structural similarity, we calculated the minimum volume enclosing ellipsoid (MVEE) for each molecule in the static form (Figs G and H in S1 File), as described earlier [58]. MVEE calculations were performed for HS chains from dimer through dodecamer. Ellipsoids based on NSGMs G2.1 and G2.2 contain an average MVEE of ~630 Å^3^, which is just slightly lower than that of HS06 (674 Å^3^ (^2^S_O_-form) or 678 Å^3^ (^1^C_4_-form)). Further, all molecules studied here presented ellipsoids that are of scalene-type (all three axial lengths are different). The longest axial lengths displayed by G2.1 and G2.2 were 15.3 and 15.5 Å, respectively, which were similar to that of HS06 in its conformational forms, ^2^S_O_ (16.7 Å) and ^1^C_4_ (15.1 Å), respectively. Likewise, the semi-minor axial lengths for the two series of molecules were also very similar (not shown). Thus, NSGMs G2.1 and G2.2 were deemed to be close mimetics of HS06 in free solution than monomer G1.1 and trimer G4.1.

**Fig 3:**
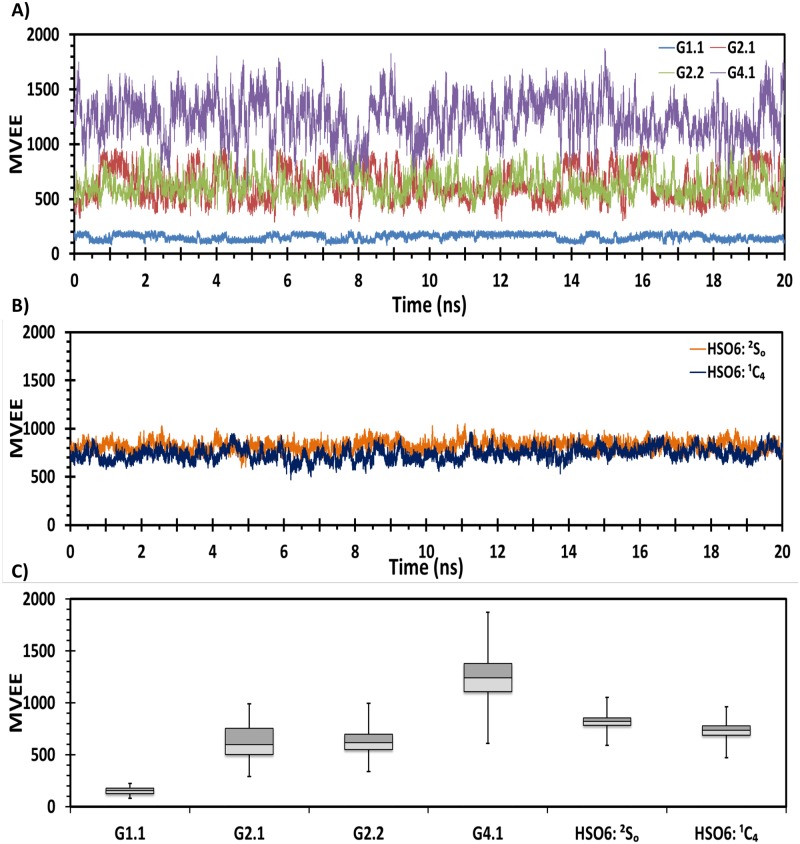
MD-based structural equivalence of NSGMs to HS06 in free solution using minimum volume enclosing ellipsoid (MVEE) as one of the comparable parameters. A) shows MVEE of each MD frame for all four NSGMs; B) shows MVEE for HS06 sequence containing IdoA2S in ^2^S_O_ and ^1^C_4_ conformations; and C) compiles the results across the MD simulation in a box plot.

To further evaluate the structural equivalence of NSGMs with HS oligosaccharides in the dynamic state, MVEE program was automated to analyze each MD ensemble for both HS06 and NSGMs (see S2 File). Fig 3 shows the fluctuation of the MVEE with time. The data show that G2.1 and G2.2 display fluctuations similar to HS06 (Fig 3A and 3B), whereas G1.1 and G4.1 present MVEE below and above, respectively, the range sampled by HS06 (Fig 3A). Quantitative comparison using the mean and range of MVEE shows that dimers G2.1 and G2.2 structurally mimic HS06, irrespective of IdoA2S pucker (Fig 3C). The free solution results suggest that majority of sulfate groups in NSGMs and GAGs are oriented equivalently in three-dimensional space (not shown).

### Equivalence of NSGMs and HS06 on binding to FGF2

A key part of assessing structural equivalence of G2.1 and G.2 with HS06 is comparison of dynamic properties in the protein-bound state. Although it is not clear how the two NSGMs exhibit their CSC inhibition properties, we reasoned that one of the growth factor receptor pathways may be targeted by NSGMs. As described earlier, several growth factor receptor pathways, including FGF–FGFR, play key roles in CSC self-renewal [53,59]. Thus, we hypothesized these ligands G2.1 and G2.2 interact with either FGF2 or with FGF–FGFR complex in a manner similar to the ligand HS06. The reason for studying the interaction with FGF as well as FGF–FGFR complex was that ligands G2.1/G2.2 may induce cell signaling modulation by regulating either FGF interaction with FGFR or by directly affecting the conformational state of FGF–FGFR complex. It is important to note that HS06 and longer HS chains bind to both FGF alone as well as FGF/FGFR1 family of proteins and initiate/modulate cell signaling [54].

Several X-ray co-crystal structure studies show that HS06 and other anionic molecules bind to FGF2 because of the presence of multiple basic residues on its surface (PDBID: 1BFC, 1BFB, 2FGF, 4FGF [29,60,61]). An overlay of these structures indicates the common region where a sulfated saccharide could bind (Fig I in S1 File). G2.1 and G2.2 were hence docked onto this heparin-binding site of FGF2 using a GOLD, a genetic algorithm-based docking tool, as described in our earlier work [62]. Each docked pose was scored using GOLD score. The best pose of G2.1 and G2.2 displayed scores of 108.6 and 109.8, respectively, which indicated good interaction in comparison to the prototypical heparin–antithrombin system (GOLD score 120–140), which is recognized as a high specificity GAG–protein system [35]. In comparison to NSGMs, the GOLD score of the best pose for HS06 with ^1^C_4_ and ^2^S_O_ forms was 100.8 and 99.3, respectively.

Although the GOLD scores appeared to show that G2.1 and G2.2 bind to FGF2 in a manner similar to HS06, the interactions were not identical. The orientation of G2.2 in the FGF2-bound form was different from that of G2.1, especially near Asn102 (Figs J and K in S1 File), which is known as the low affinity region [29,63]. To better elucidate the recognition profiles, we studied dynamical behavior of each co-complex in a box of explicit water molecules.

**Fig 4:**
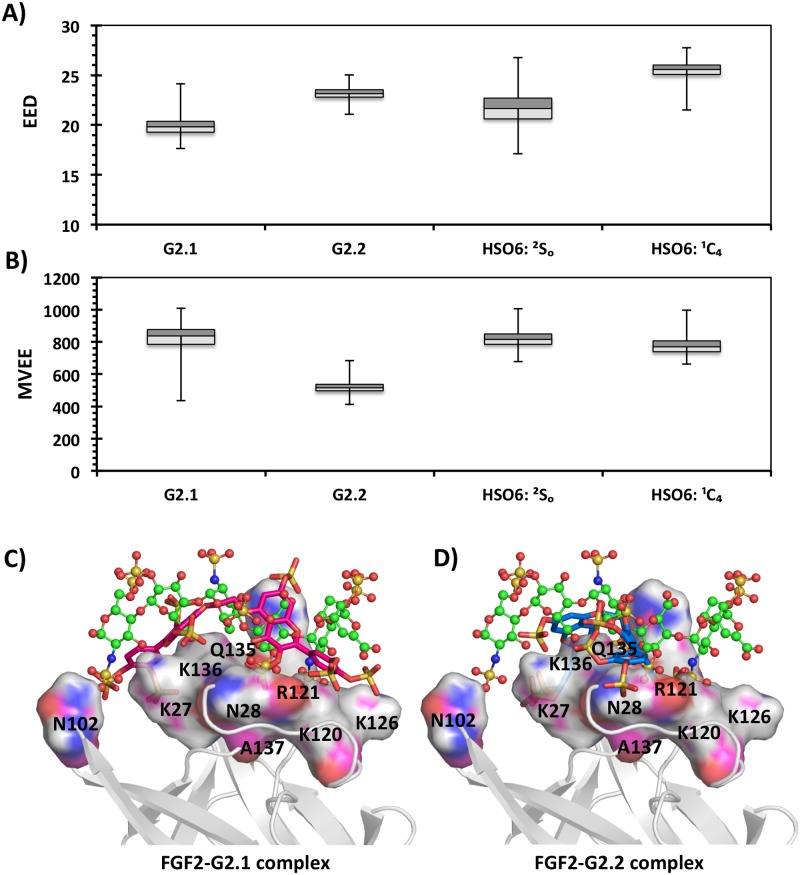
Comparison of NSGMs and HS06 bound to FGF2 in the acidic binding pocket using EED and MVEE as parameters. The spatial equivalence of NSGMs to HS06 sequences containing IdoA2S in ^2^S_O_ and ^1^C_4_ conformations was evaluated from the EED (A) and MVEE (B) calculated for each MD frame in FGF2 bound form. The orientation of NSGMs (G2.1-pink, G2.2-blue, stick representation) and HS06 (green, ball & stick representation) in FGF2 binding pocket with interacting residues (single letter code) colored by atom-type.

MD simulations for FGF2–G2.1 and FGF2–G2.2 co-complexes showed an overall stable structure with minimal root mean square deviation (RMSD) from the average structures (only 1.01 Å and 1.02 Å, respectively). Likewise, the two HS06–FGF2 co-complexes displayed deviations in the similar range (Fig L in S1 File). In a manner similar to the parameters used for studying equivalence of NSGMs and HS06 in free form, we calculated the EED and MVEE in the bound form (Fig 4A and 4B, and Fig M in S1 File). The results show that both NSGMs marginally differ in these parameters when compared to the bound HS06. The small differences appear to arise from the differences in the orientation of G2.1 and G2.2 when compared to that of HS06 as shown by the x-ray crystal structure of the co-complex (Fig 4C and 4D).

**Fig 5:**
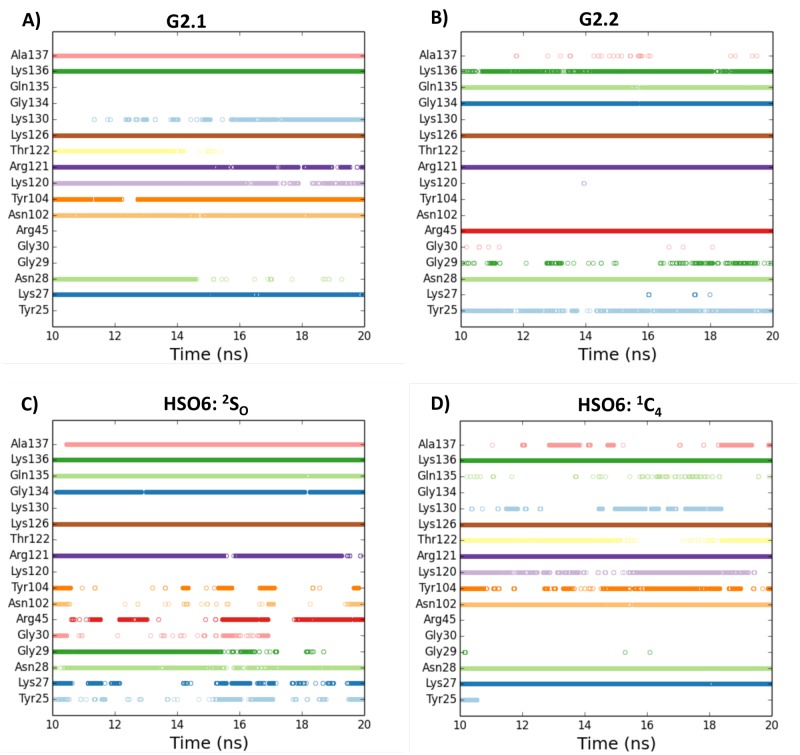
The consistency of intermolecular hydrogen bonds across the MD simulation (final 10 ns) for FGF2 residues. The occurrence of inter-molecular hydrogen bonds between FGF2 residues (shown on y-axis) and small molecule ligands (NSGMs and HS06) are shown for each frame of the final 10 ns of the MD run. A) FGF2–G2.1 complex; B) FGF2–G2.2 complex; C) FGF2–HSO6(^2^S_O_) complex; D) FGF2–HS06(^1^C_4_) complex.

To further parse the similarities and differences, hydrogen bond (H-bond) formation was evaluated by the VMD tool using a donor-acceptor distance cutoff of 3.5 Å and angle cutoff of 60°, as described earlier [33]. Fig 5 shows the consistency of H-bonding between FGF2 and ligands for the final 10 ns of simulations that display highest level of co-complex stability. The analysis shows that some H-bonds, e.g. with Arg121, Lys126 and Lys136, occur in nearly every frame for G2.1, G2.2 as well as HS06 highlighting similarity of interactions. By contrast, other H-bonds are consistent only for one or two of the three molecules, e.g., Arg45 for G2.2, or Gln135 and Asn28 for G2.2 and HS06, or Asn102 for G2.1 and HS06 (Fig 5), suggesting microscopic differences in interactions.

A quantitative analysis of the ability to form intermolecular H-bonds was deduced by calculating the percent H-bond occupancy, which is the proportion of time an amino acid residue forms H-bond with its ligand (Fig N in S1 File). The analysis revealed that Lys136 contributed most H-bonds for G2.1, whereas it was Arg121 for G2.1. Similar analysis for the dynamics of HS06–FGF2 co-complex shows that both Arg121 and Lys136 contribute nearly equal H-bond occupancy.

Another tool to assess structural equivalence is the dynamism of ligands in the protein bound state. When RMSD from the average structure of a ligand at each time frame was calculated, G2.1 displayed an RMSD of ~3 Å whereas it was <2 Å for G2.2 (Fig O in S1 File). K-means clustering of conformations occurring in the bound state showed that G2.1 sampled 5 clusters, whereas G2.2 sampled only one cluster (Fig O in S1 File) [34]. This implied that although G2.2 and G2.1 interacted with similar set of residues, the motion of G2.2 within the site of binding was much more restricted than G2.1.

Finally, we calculated the theoretical free energy of binding (ΔG) using MM/PB(GB)SA method implemented in Ambertools14 [36]. The binding energies calculated for FGF2–G2.1 and FGF2–G2.2 co-complexes were -73.5±10.0 and -68.6±5.8 kcal/mol, respectively. These binding energies are in line with that of the HS06, which displayed -74.0±11.1 and -67.3±9.4 kcal/mol for IdoA2S in ^2^S_O_ and ^1^C_4_ forms, respectively and these values are in agreement with recent computational studies on HS06 [64]. Yet, the overall similarity in binding energies does not imply identical interactions at individual residue level. For example, Lys126 (-12.0±3.7 kcal/mol) and Arg121 (-11.1±1.4 kcal/mol) were identified as the prime contributors of affinity for G2.1 and G2.2, respectively, whereas Gln135 (-10.2±1.6 kcal/mol) and Arg121 (-9.0±2.8 kcal/mol) were the dominant residues for HS06 in the ^2^S_O_ and ^1^C_4_ forms, respectively (Fig P in S1 File). In addition, differences in binding energy contributions from several other residues were evident between NSGMs and HS06 (e.g., Lys120, Gln135).

### Equivalence of NSGMs and HS06 on binding to FGF2–FGFR1 complex

It is well established that the receptor FGFR1 dimerization is a decisive step in FGF2–FGFR1 signaling and HS proteoglycan plays an important role in this action by engineering a ternary complex in the extracellular matrix [65]. The crystal structure of the FGF2–heparin–FGFR1 ternary complex shows a canyon in which the anionic molecule binds [30,57]. We studied dynamics of G2.1 and G2.2 bound to this region to assess their structural equivalence with HS06.

**Fig 6:**
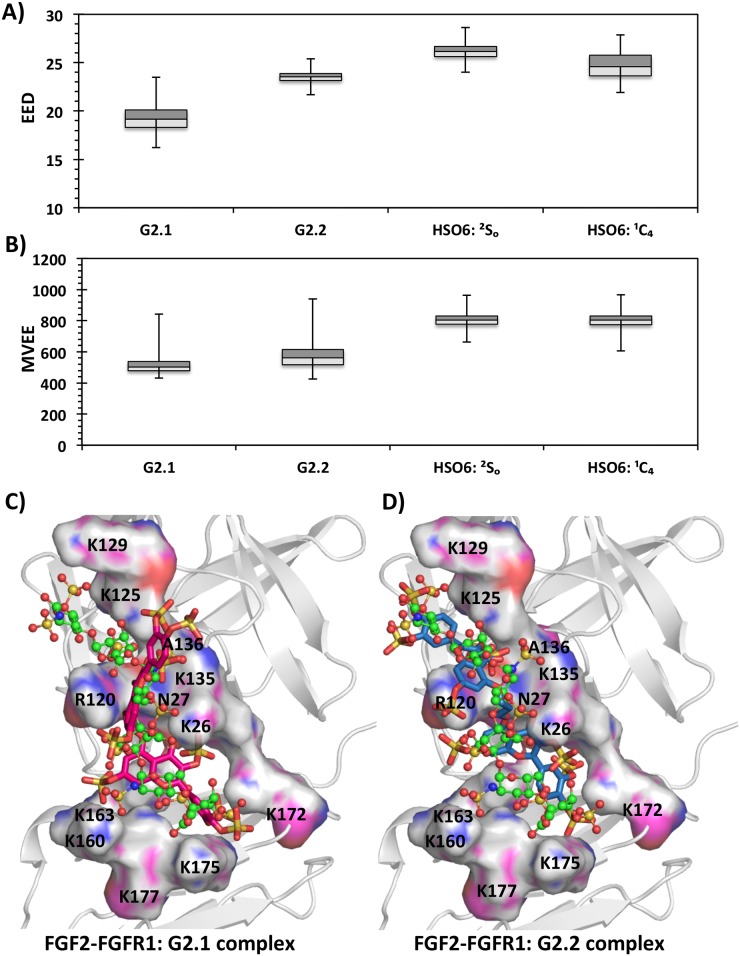
Comparison of NSGMs and HS06 bound to FGF2–FGFR1 using EED and MVEE as parameters. The spatial equivalence of NSGMs to HS06 sequences containing IdoA2S in ^2^S_O_ and ^1^C_4_ conformations was evaluated from the EED (A) and MVEE (B) calculated for each MD frame in FGF2–FGFR1 bound form. The orientation of NSGMs (G2.1-pink, G2.2-blue, stick representation) and HS06 (green, ball & stick representation) in FGF2 binding pocket with interacting residues (single letter code) colored by atom-type.

Docking studies were performed for G2.1, G2.2 and HS06 in a manner described above for FGF2 using the 1FQ9 crystal structure [30]. The GOLD scores for G2.1 and G2.2 ternary complex with FGF2–FGFR1 were 119.5 and 144.7, respectively. The GOLD scores for HS06 in ^1^C_4_ and ^2^S_O_ forms were 113.7 and 122.3, respectively (Fig K in S1 File). Analysis of binding poses suggested geometries that were similar for both NSGMs (Fig J in S1 File). Following docking, we performed explicit MD simulations for the FGF2–FGFR1 ternary complexes with the two NSGMs and HS06. In all four simulations, the protein exhibited equivalent RMSD fluctuations with respect to corresponding average structure (Fig Q in S1 File). Calculation of the EED and MVEE in the bound form indicated that G2.2 more closely matches HS06 (either forms) than G2.1 in interacting with FGF2–FGFR1 binary complex (Fig 6A and 6B) and (Fig M in S1 File). This is further supported by the almost identical orientation of NSGMs G2.1 and G2.2 in the binding cavity as that of HS06 (Fig 6C and 6D).

**Fig 7:**
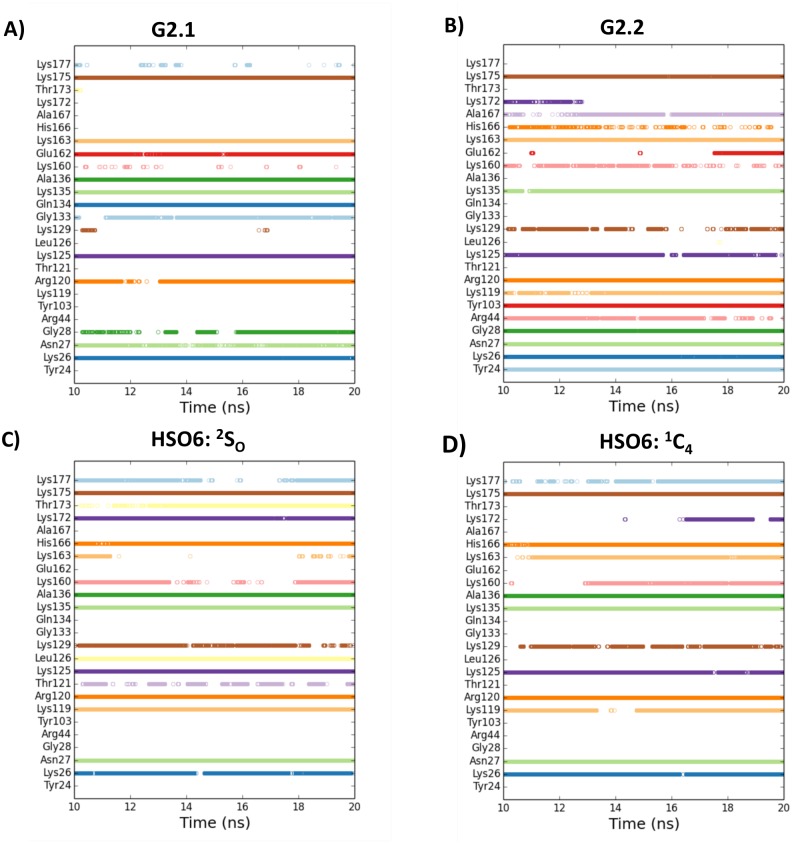
The consistency of intermolecular hydrogen bonds across the MD simulation (final 10 ns) for FGF2–FGFR1 residues. The occurrence of inter-molecular hydrogen bonds between FGF2–FGFR1 residues (shown on y-axis) and small molecule ligands (NSGMs and HS06) are shown for each frame of the final 10 ns of the MD run. A) FGF2–FGFR1–G2.1 complex; B) FGF2–FGFR1–G2.2 complex; C) FGF2–FGFR1–HSO6(^2^S_O_) complex; D) FGF2–FGFR1–HS06(^1^C_4_) complex.

We also evaluated the inter-molecular H-bond formation for each ternary co-complex. Fig 7 shows consistency of H-bonds across 10 to 20 ns (10,000 frames) of MD experiments. At a qualitative level, both G2.1 and G2.2 forms multiple stable H-bonds throughout the simulation in a manner similar to HS06. For example, Lys26, Asn27, Arg120, Lys125, Lys135, and Lys175 form fairly consistent H-bonds with all three molecules [30,66]. These interactions span almost the entire canyon formed by the two interacting proteins. Differences do arise including G2.2 interacting well with additional residues such as Ala167, Tyr103, Arg44, and Gly28, while G2.1 not displaying comparatively equal consistent H-bonds (Fig 7). At a quantitative level, the H-bond occupancies confirm the above interactions (Fig R in S1 File). In combination, MD studies show that FGFR1 binding region was more important for G2.2 recognition than G2.1 (Fig S in S1 File). With respect to fluctuations in the bound form, G2.1 displayed three prominent clusters in the ternary complex, which is much less than that noted for FGF2 binary complex. In contrast, G2.2 gave a single cluster (Fig S in S1 File), as also observed with FGF2 alone.

Finally, calculation of the ΔG for 1,000 MD structures spaced 10 ps apart from 10 ns to 20 ns for the ternary complex showed energies of -95.0±8.9 kcal/mol and -116.7±12.0 kcal/mol for G2.1 and G2.2, respectively. This implies that addition of a CH_2_ linker in G2.2 contributed ~22 kcal/mol more energy than G2.1. Likewise, HS06 displayed energies of -130.7±11.9 kcal/mol and -144.3±14.3 kcal/mol for IdoA2S in the ^2^S_O_ and ^1^C_4_ forms, respectively. At an individual residue level, Lys163 (-14.9±2.2 kcal/mol) and Arg120 (-16.1±3.3 kcal/mol) contributed most for binding to G2.2, whereas Lys175 (-7.1±1.9 kcal/mol) and Lys135 (-12.7±1.6 kcal/mol) were important for G2.1. It is important to note that these key residues belong to FGFR1 and FGF2 binding regions, which should play key role in organization of the ternary complexes (Fig T in S1 File). The simulations for HS06 show that irrespective of the IdoA2S puckering, Lys135 and Lys125 from the FGF2 binding region and Lys163 and Lys160 as from the FGFR1 binding region contribute the most energy (Fig T in S1 File).

## Concluding compilation of results–development of algorithm

**Fig 8:**
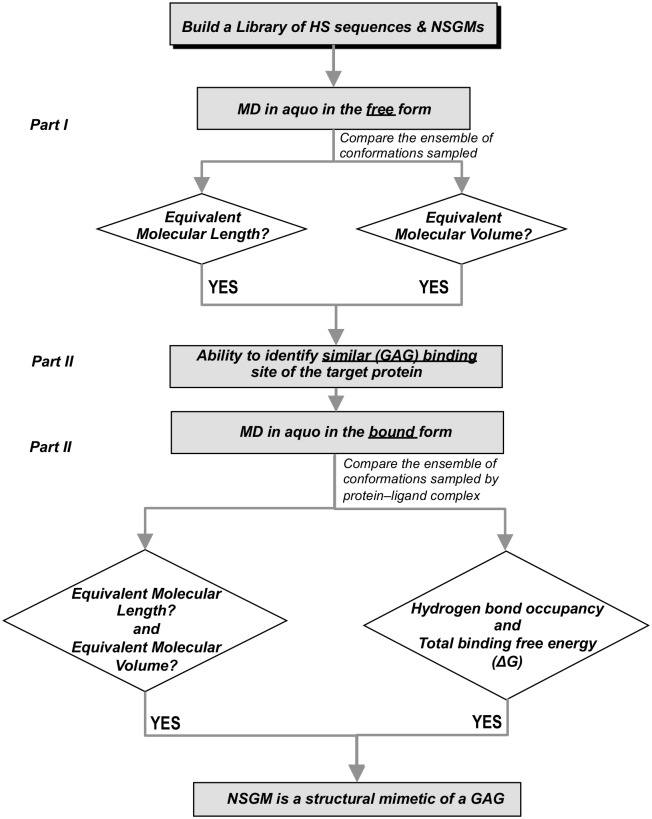
Overview of the algorithm developed for assessing GAG structure mimicking potential of NSGMs. We have developed an algorithm consisting of three sequential legs. The first leg is to identify GAG chain length mimicking potential in free solution using end-to-end distance (EED) and minimum volume enclosing ellipsoid (MVEE) as two parameters. The second leg identifies whether the desired NSGMs bind to the same protein binding site as the target GAG. The third leg attempts assessing the mimicking potential in the protein-bound form with explicit solvent MD simulations. In this comparison is made to GAG sequence using EED, MVEE, inter-molecular hydrogen bonds and the total binding free energy parameters.

The above MD studies in free and protein-bound states can now be compiled in the form of an algorithm that aids evaluation of structural equivalence of NSGMs and GAGs. Our studies can be described in terms of a generic three-step MD-based algorithm (Fig 8). In the first step, parameters related to the range of conformations sampled by an NSGM and a target GAG in water should be compared. In the second step, the similarity of their binding site geometries should be evaluated. In the final step, conformational dynamics and atomic interactions in the protein-bound state should be evaluated. This algorithm is completely different from the simplistic comparison involving overlap of sulfate groups in the static state, which has been traditionally employed in the design/development of NSGMs [7,16,17,39].

In terms of results on NSGMs and HS06 studied here, the free solution MD of four NSGMs demonstrated an extended nature of the dimeric agents G2.1 and G2.2, which compared favorably with HS06 in terms of EED and MVEE (Figs 2C and 3C). Both molecules were essentially equivalent to HS06 in terms of free solution dynamics. By contrast, G1.1 and G4.1 could be completely ruled out. In the second leg of the algorithm, we utilized two target proteins FGF2 and FGFR1. G2.1 and G2.2 were docked onto the anionic binding site of the two proteins, which demonstrated binding geometries that were reasonably similar to that of HS06.

**Fig 9:**
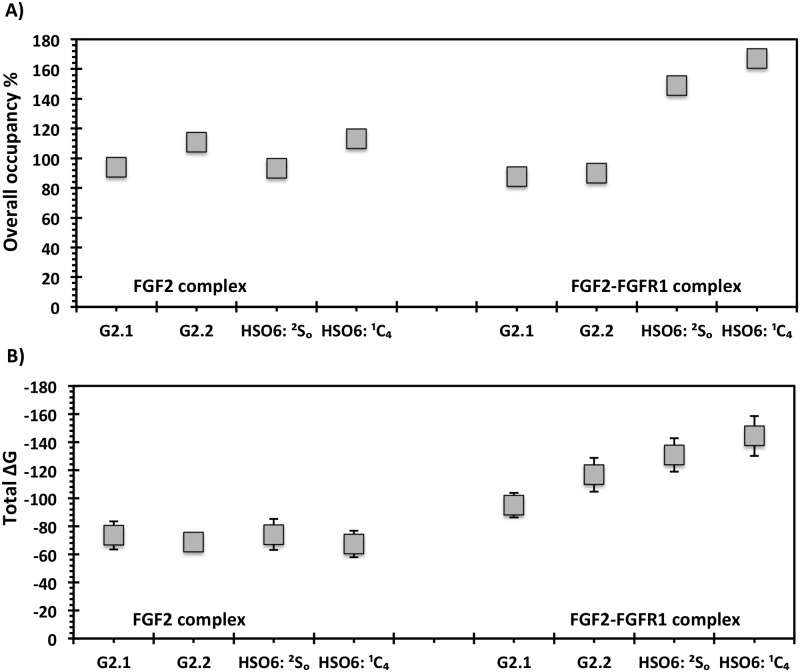
Comparison of NSGMs and HS06 in the protein bound state using overall average inter-molecular hydrogen bond occupancy (A) and total binding free energy (B). A) shows the stability of bound NSGMs and HS06 based on average residue-level, inter-molecular hydrogen bond occupancy; B) shows the stability of the bound NSGMs and HS06 based on total binding free energy ΔG (in simulated kcal/mol, error bars shows standard deviation). See text for details.

In the third leg, MD starting with the most optimal geometries observed in the second leg was performed. In studies with FGF2, whereas the EED more closely resembled HS06 for G2.2 (Fig 4A), the MVEE equivalence was better for G2.1 (Fig 4B). The similarity of these two parameters did not automatically equate to similarity in interactions with residues of the binding site. In fact, many residue level interactions were found to be different. To quantify these differences, the overall average H-bond occupancies were calculated. Interestingly, using this parameter G2.2 was found to be closer to the ^1^C_4_ form of HS06 (Fig 9A), whereas G2.1 was more aligned with ^2^S_O_ form of HS06 (Fig 9A). Likewise, the overall ΔG of interaction showed that both G2.2 and G2.1 were similar to HS06 in the FGF2-bound state (Fig 9B). Similar analysis for complexes with FGF2–FGFR1 demonstrated that both G2.1 and G2.2 could simultaneously bind FGF2 and FGFR1 in the co-complex in the manner of HS06. In terms of EED and MVEE, G2.2 was slightly better at structurally mimicking HS06 than G2.1 (Fig 6A and 6B). Yet, notable differences in atomic interactions with the FGF2–FGFR1 co-complexes were observed. Overall, the H-bond occupancies for G2.2 and G2.1 were similar but lower than that for HS06 (Fig 9A). Likewise, overall ΔG calculation showed that G2.2 was closer to mimicking HS06 in comparison to G2.1 (Fig 9B). In Because FGF2–FGFR1 co-complex is likely to be the key signal transduction mediator with respect to CSC function, we give more weightage to results on this protein target. Thus, these results imply that G2.2 could be expected to mimic HS06 better than G2.1. In turn, these two NSGMs are much better than G1.1 and G4.1.

## Significance

Our success with selective inhibition of CSCs, but not cancer bulk cells [11], by G2.2 and G2.1 from a library of 53 NSGMs prompted us to posit that the fine structure of these molecules was mimicking the action of heparin/heparan sulfate. *A priori* the molecule mechanism of CSC inhibition by HS oligosaccharides has not been elucidated as yet, although we have recently identified that HS06, but not shorter or longer HS sequence, is the optimal chain length for inhibition [55]. In this work, we has also elucidated that CSC inhibition by HS06 is mediated by activation of p38 MAPK [55]; however, how the signal is transduced from the cell exterior to MAPK activation remains to be deciphered. Thus, our hypothesis that G2.2 and G2.1 preferentially bind FGF2 and/or FGF2–FGFR1 is just one of the many growth factor/growth factor receptor (GF/GFR) related possibilities. Other GF/GFR pathways that could be targeted by G2.2 and/or G2.1 include EGF/EGFR, HGF/HGFR, and others, which have also been implicated in CSC growth and differentiation processes [67,68]. The focus of this work was not elucidation of mechanism of action of NSGMs and/or HS06 by comprehensively screening all such pathways. Our focus was much more limited. Considering that NSGMs had been found to functionally work as GAG mimetics in a number of cases [7,9,13,16], we wanted to assess whether NSGMs are structurally equivalent to distinct sequences of HS. As a test case, we selected CSC inhibition by G2.2 and G2.1, but not by G1.1 and G4.1 [11], and develop an algorithm to more comprehensively test for structural equivalence in recognition of putative protein targets.

This work shows that structural equivalence of a NSGM and GAG in the static state may be useful but is likely to insufficient. We propose that evaluation should be performed in two dynamic states–the free and the bound–to more comprehensively assess structural equivalence. We developed an algorithm based on quantitative analysis of the dynamic ensembles of free and bound states and incorporated similarity of recognition and interactions in the bound state. This algorithm can be automated to elucidate the rank order of GAG mimicking potential of NSGM-like molecules. We also propose that it may be possible to elucidate the preferred protein binding targets of specific NSGMs. These studies are being performed and will be communicated in due course.

Finally, the major significance of this work is to help identify and/or design lead NSGM(s) that mimic the action of synthetically inaccessible, heterogeneous natural GAG sequences, which may be discovered in the future to modulate biological functions. Thus the algorithm contributes to the continuing efforts of developing clinical relevant candidate agents based on highly sulfated, non-saccharide scaffolds [5].

## Supporting information

S1 File This file contains 20 supporting figures from Fig A to Fig T. (PDF)

S2 File Automated code for measuring minimum volume enclosing ellipsoid (MVEE). (PDF)
