# Supplementary material for: A molecular dynamics-based algorithm for evaluating the glycosaminoglycan mimicking potential of synthetic, homogenous, sulfated small molecules
Source: PLoS One. 2017 Feb 9;12(2):e0171619. doi: 10.1371/journal.pone.0171619 (PMC5300208; doi:10.1371/journal.pone.0171619)
Supplement: S1 File — (PDF) [file pone.0171619.s001.pdf]

Supplementary information for

A Molecular Dynamics-based Algorithm for Evaluating the Glycosaminoglycan Mimicking Potential of Synthetic, Homogenous, Sulfated Small Molecules

Balaji Nagarajan,[1] Nehru Viji Sankaranarayanan,[1] Bhaumik B. Patel,[2,3] and Umesh R. Desai[1,*]

[1]*Institute for Structural Biology, Drug Discovery and Development and Department of Medicinal Chemistry, Virginia Commonwealth University, Richmond, VA*

[2]*Hunter Holmes McGuire VA Medical Center, Richmond, VA 23249*

[3]*Division of Hematology, Oncology, and Palliative Care, Department of Internal Medicine and Massey Cancer Center, Virginia Commonwealth University, Richmond, VA 23298*

*Address for correspondence: Dr. Umesh R. Desai, 800 E. Leigh Street, Suite 212, Richmond, VA 23219. Ph (804) 828-7328; Fax (804) 827-3664; urdesai@vcu.edu

Table of Contents

#	Title	Pg.
1	S1 Fig. Heparin hexasaccharide (HS06, PDBID 1HPN)	3
2	S2 Fig. Number of clusters	4
3	S3 Fig. Non-hydrogen atoms overlay of lowest energy structure with cluster centroid	5
4	S4 Fig. Definition of End-to-end distance (EED)	6
5	S5 Fig. Distribution of end-to-end distance (EED) for NSGMs.	7
6	S6 Fig. The principal component analysis (PCA)	8
7	S7 Fig. Definition of minimum volume enclosing ellipsoid (MVEE)	9
8	S8 Fig. Distribution of minimum volume enclosing ellipsoid (MVEE) for NSGMs	10
9	S9 Fig. Overlay of experimental X-ray co-crystal structures of FGF2	11
10	S10 Fig. Molecular docking of NSGMs in the heparin-binding region of FGF2 (1BFC) and FGF2-FGFR1 (1FQ9) complex using GOLD docking tool	12
11	S11 Fig. Molecular docking of HSO6 in the heparin-binding region of FGF2 (1BFC) and FGF2-FGFR1 (1FQ9) complex using GOLD docking tool	13
12	S12 Fig. RMSD of FGF2-NSGMs and FGF2-HSO6 complex	14

Table of Contents

#	Title	Pg.
13	S13 Fig. Distribution of end-to-end distance (EED) and minimum volume enclosing ellipsoid (MVEE) of NSGMs in protein bound form for both FGF2 and FGF2-FGFR1.	15
14	S14 Fig. Intermolecular-hydrogen bond occupancy for the FGF2 complex	16
15	S15 Fig. The conformational flexibility of FGF2 bound dimers	17
16	S16 Fig. Single residue energy decomposition of FGF2 complexes	18
17	S17 Fig. RMSD of FGF2-FGFR1 NSGMs and FGF2-FGFR1 HSO6 complex	19
18	S18 Fig. Intermolecular-hydrogen bond occupancy for the FGF2-FGFR1 complex	20
19	S19 Fig. The conformational flexibility of FGF2-FGFR1 bound NSGMs dimers	21
20	S20 Fig. Single residue energy decomposition of FGF2-FGFR1 complexes	22

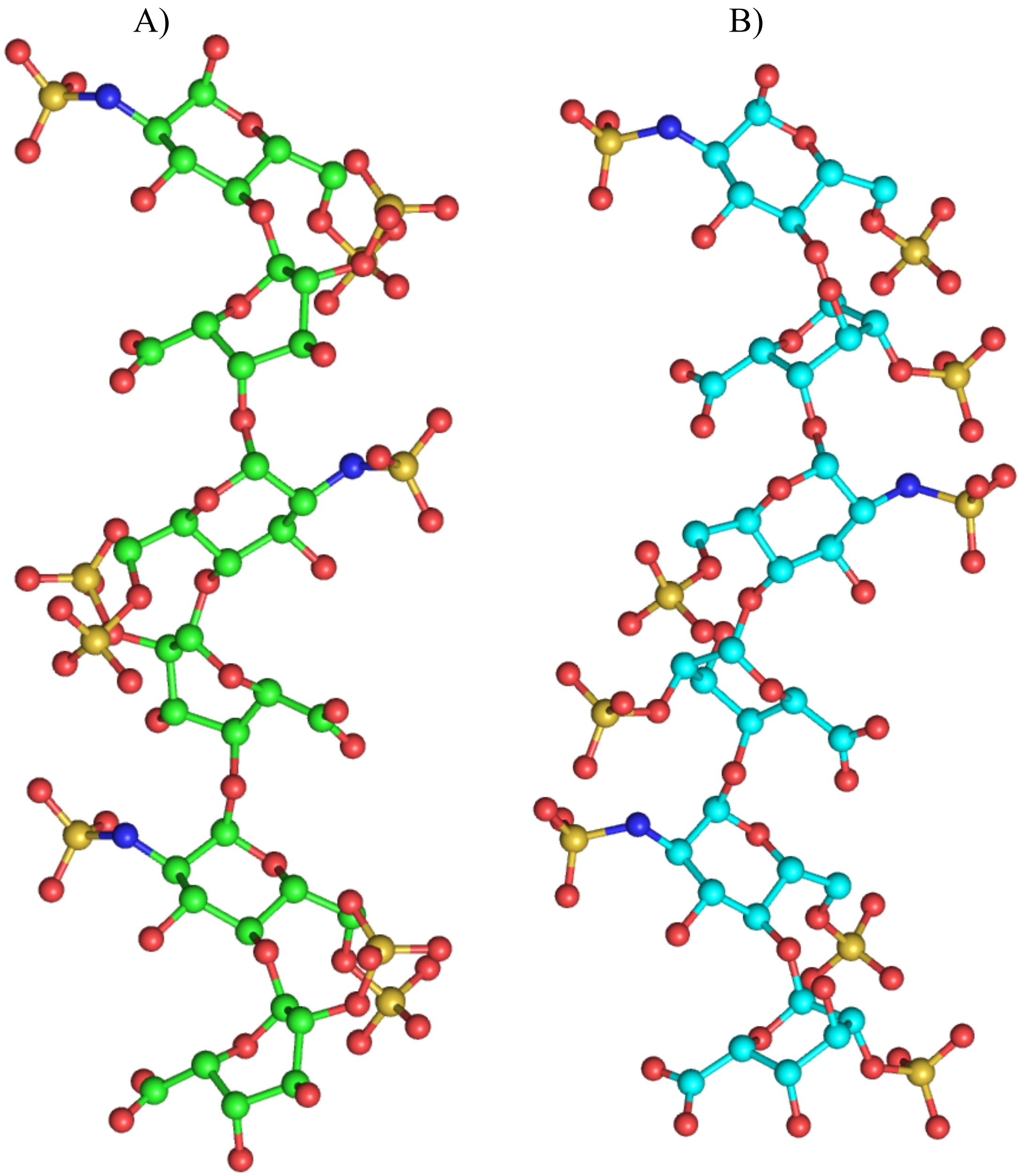

S1 Fig. Heparin hexasaccharide (HS06 PDBID 1HPN).

Shows the initial starting structure of HSO6 for free solution molecular dynamics simulation, taken from the N.M.R. structure (PDBID: 1HPN). A) HSO6 with IdoA(2S) in skew boat $^{2}S_{O}$ conformation, B) HSO6 with IdoA(2S) in chair $^{1}C_{4}$ conformation.

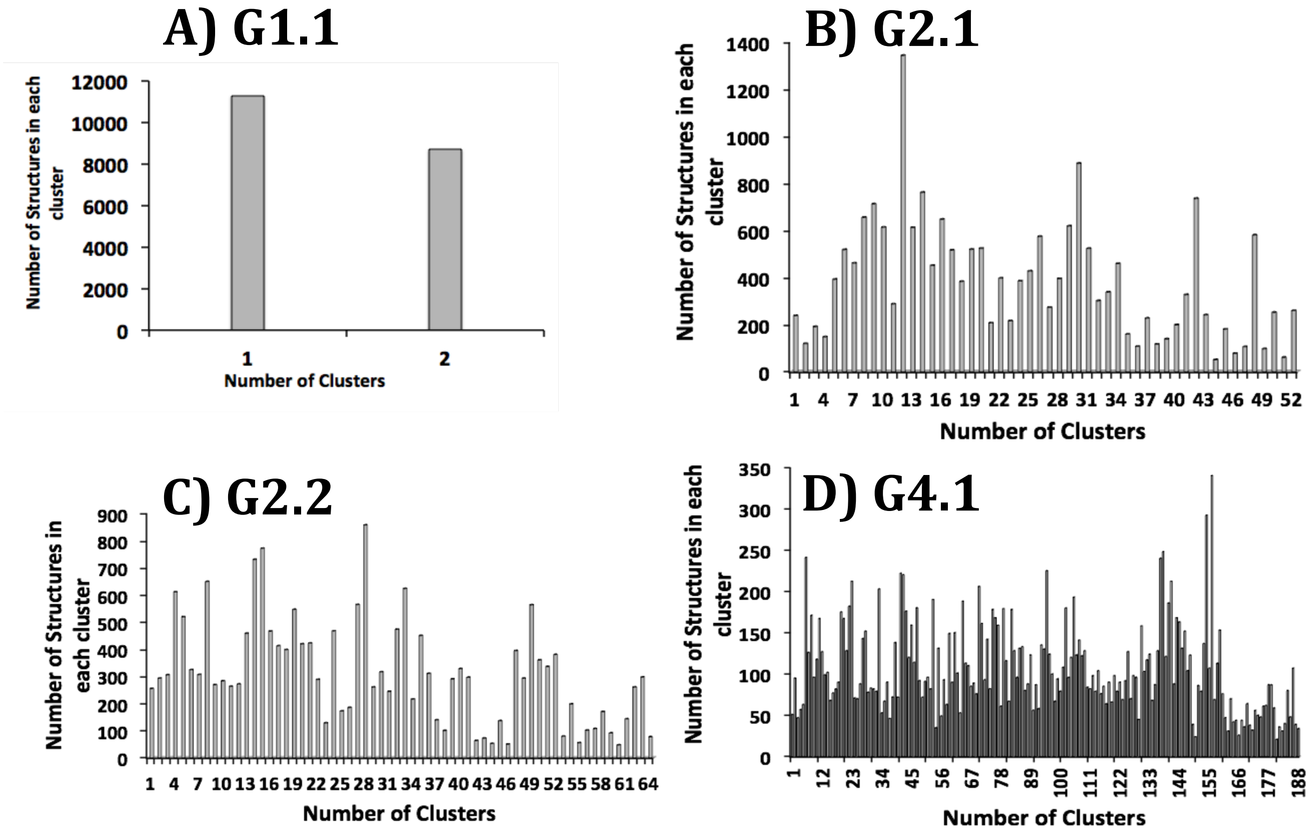

S2 Fig. Number of clusters.

Clusters calculated using K-means algorithm from the MD simulated ensemble of structures based on RMSD A) monomer G1.1 B) dimer G2.1 C) dimer G2.2 D) trimer G4.1.

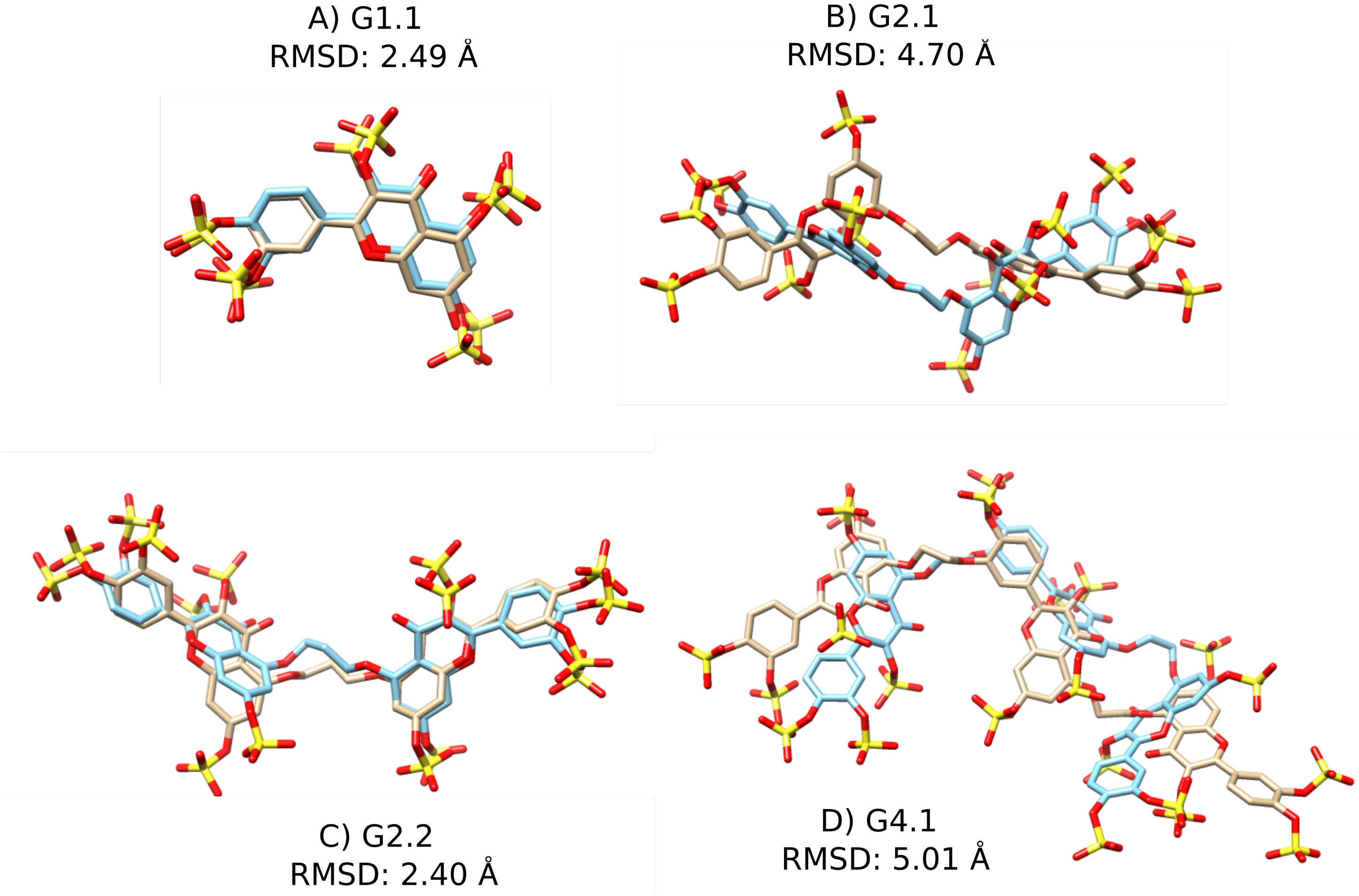

S3 Fig. Non-hydrogen atoms overlay of lowest energy structure with cluster centroid.

Superposition of the lowest energy structure to the representative structure from the largest cluster A) monomer G1.1 B) dimer G2.1 C) dimer G2.2 D) trimer G4.1.

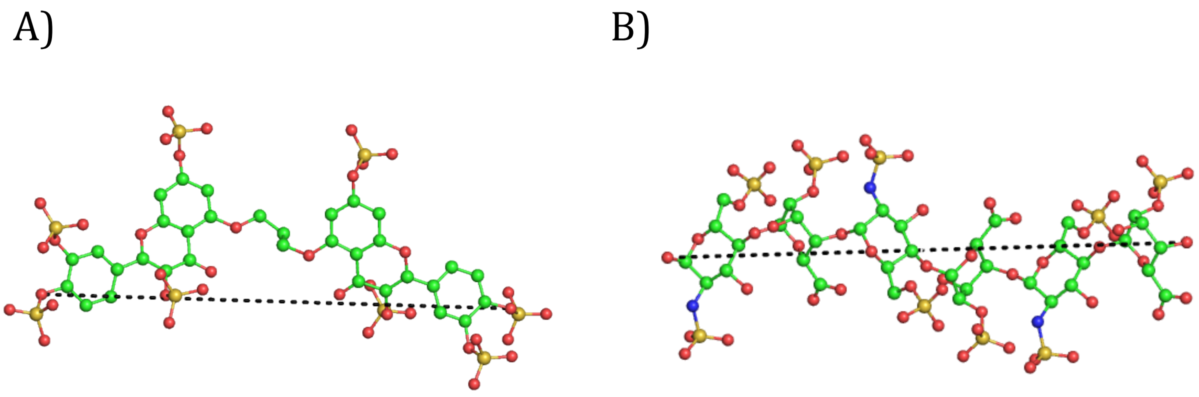

S4 Fig. Definition of End-to-end distance (EED).
A) Measured from the furthest oxygen atoms, shown for NSGM dimer G2.2 B) Measured from the furthest oxygen atoms, shown for HSO6: $^{1}C_4$

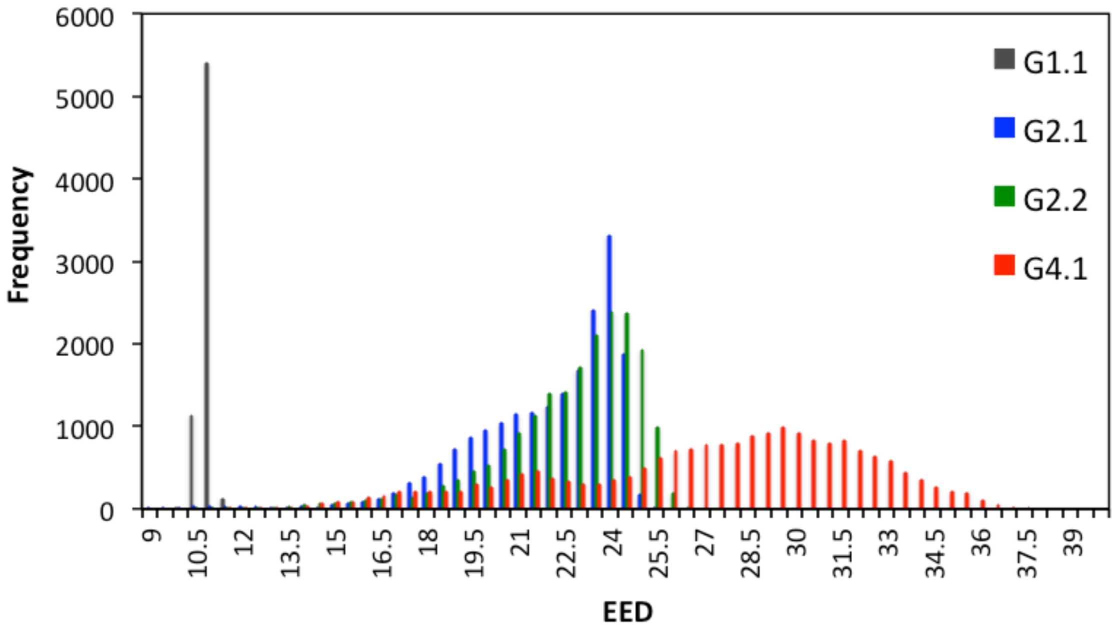

S5 Fig. Distribution of end-to-end distance (EED) for NSGMs.
Distribution of EED (in Å) for NSGMs G1.1 (The actual value is 3 times the shown value), G2.1, G2.2 and G4.1. The distribution clearly shows that G1.1 and G4.1 are not in the range of the EED of HSO6 (26.4 Å (IdoA2S in $^{2}S_{O}$-form) or 26.1 Å (IdoA2S in $^{1}C_{4}$-form)). While G2.1 and G2.2 are in range with HSO6 (since the molecule of our choice should have approximately equal length but should not exceed HSO6).

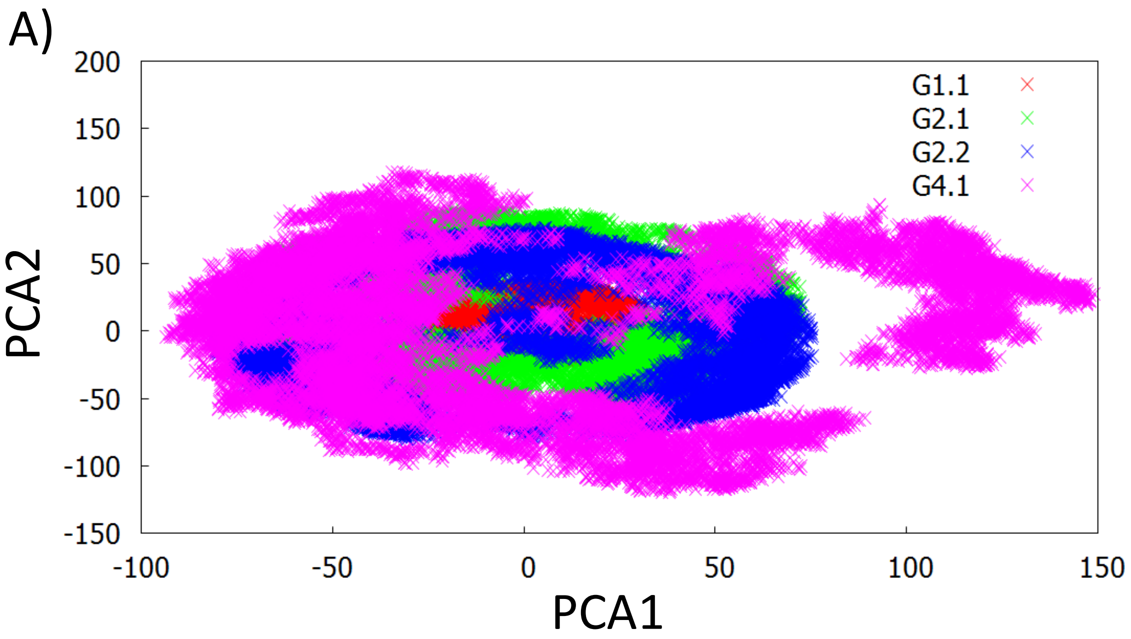

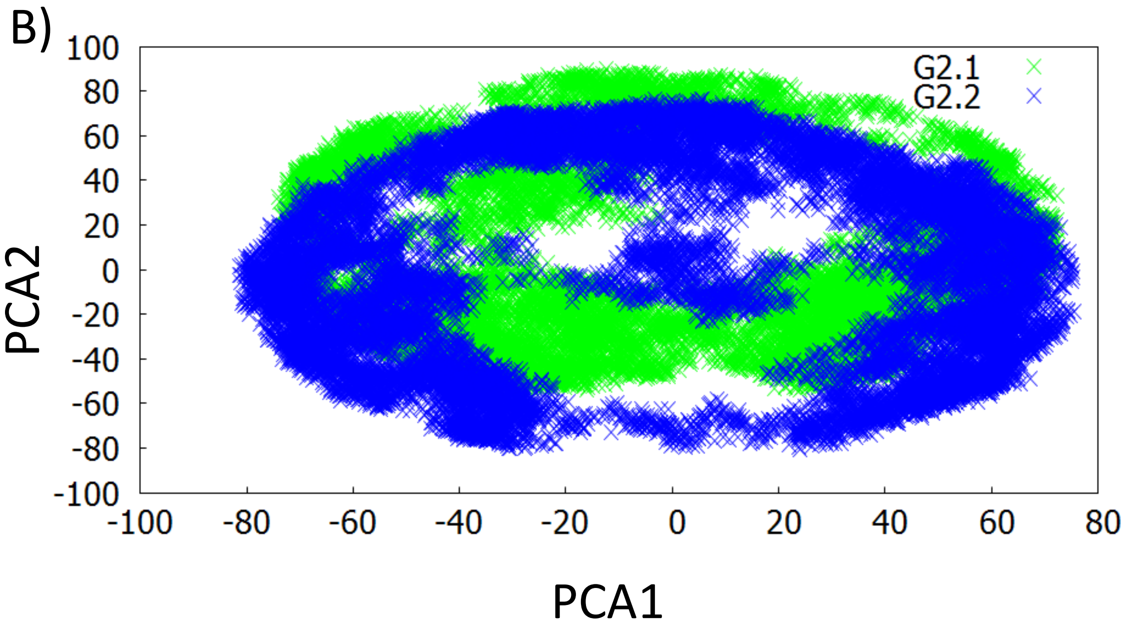

S6 Fig. The principal component analysis (PCA).
Obtained from the projection of multidimensional space to appropriate low dimension space. Distribution of the molecules over the first two primary axes pca1 and pca2 are shown, A) for all four NSGMs (G1.1, G2.1, G2.2 and G4.1), B) for two dimers G2.1 and G2.2.

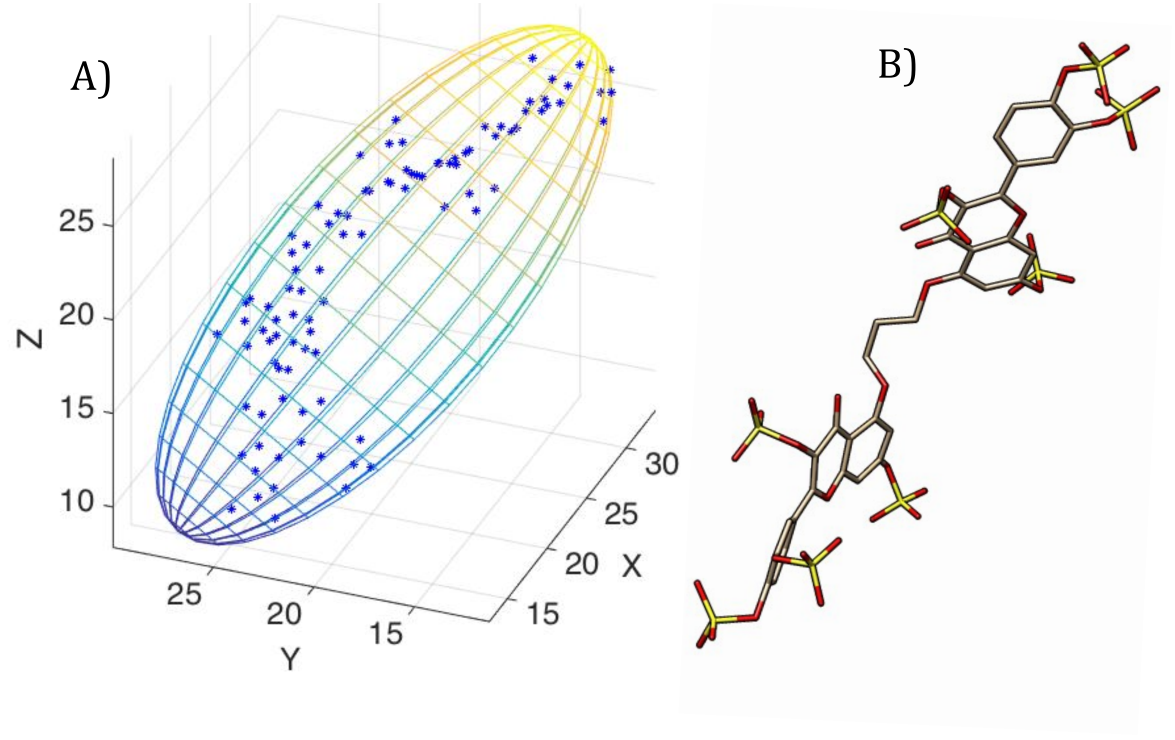

S7 Fig. Definition of minimum volume enclosing ellipsoid (MVEE).
A) Co-ordinate space of dimer G2.2 (enclosed blue dots represents each atom of G2.2), B) the molecule enclosed inside the minimum volume ellipsoid (for automated algorithm see S2 File).

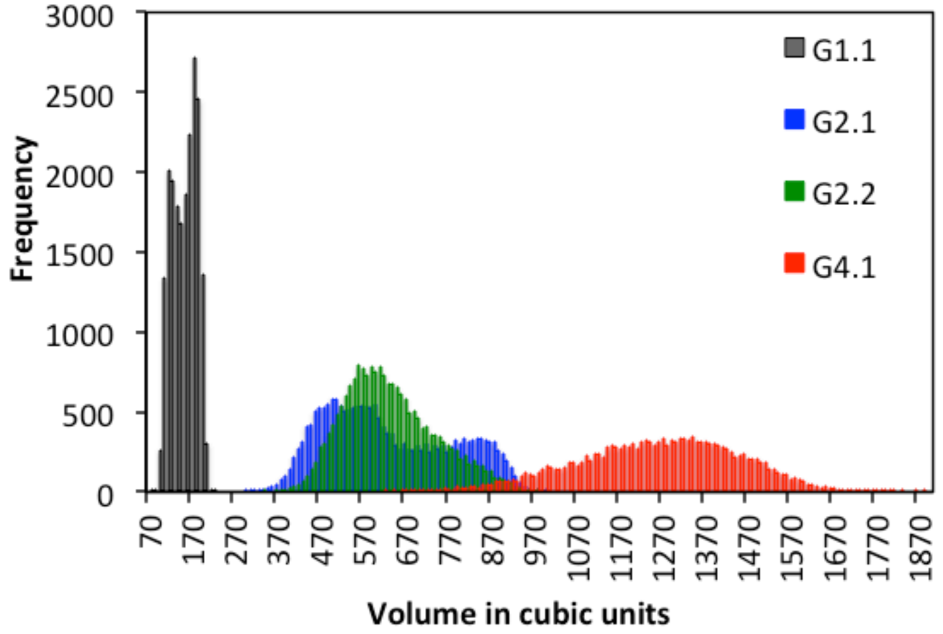

S8 Fig. Distribution of minimum volume enclosing ellipsoid (MVEE) for NSGMs. The frequency distribution of the MVEE for NSGMs G1.1, G2.1, G2.2 and G4.1. The distribution clearly shows that G1.1 and G4.1 are not in the range of the volume of HSO6 (674 Å^3 (IdoA2S in ^2S_O-form) or 678 Å^3 (IdoA2S in $^1C_4$-form)). While G2.1 and G2.2 are in range with HSO6.

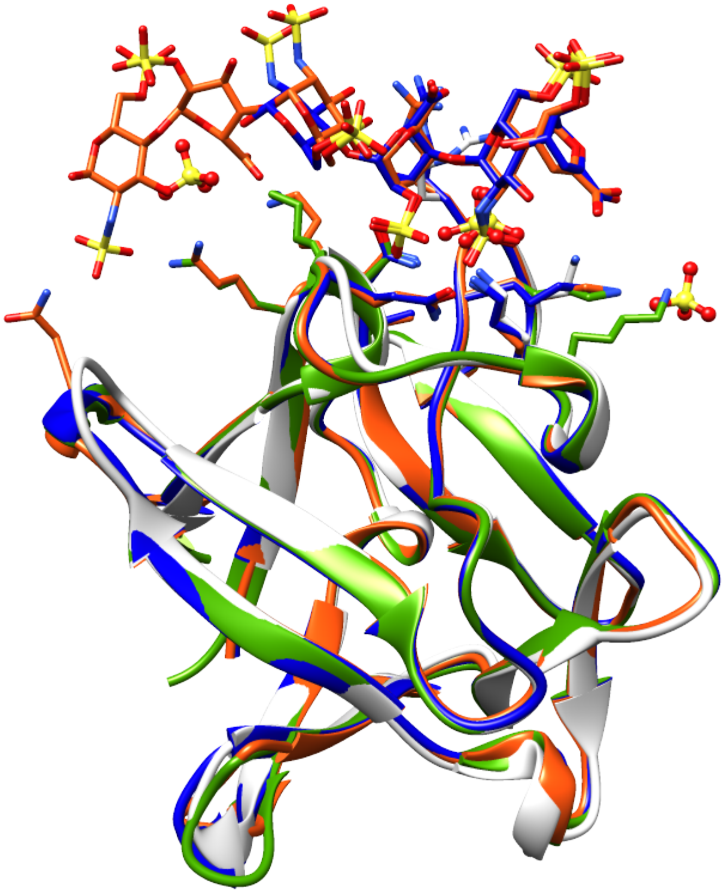

S9 Fig. Overlay of experimental X-ray co-crystal structures of FGF2.
The published crystal structures with heparin tetra (PDB ID: 1BFB in blue color), hexasaccharide (PDB ID: 1BFC in orange color) shown as stick representation and with other anionic molecules (shown in ball and stick representation) (PDB ID: 2FGF in green color & 4FGF in white color). The over all structure superposition shows a RMSD of less than 0.7 Å with respect to one another, with consistent surface-binding residues from the protein FGF2.

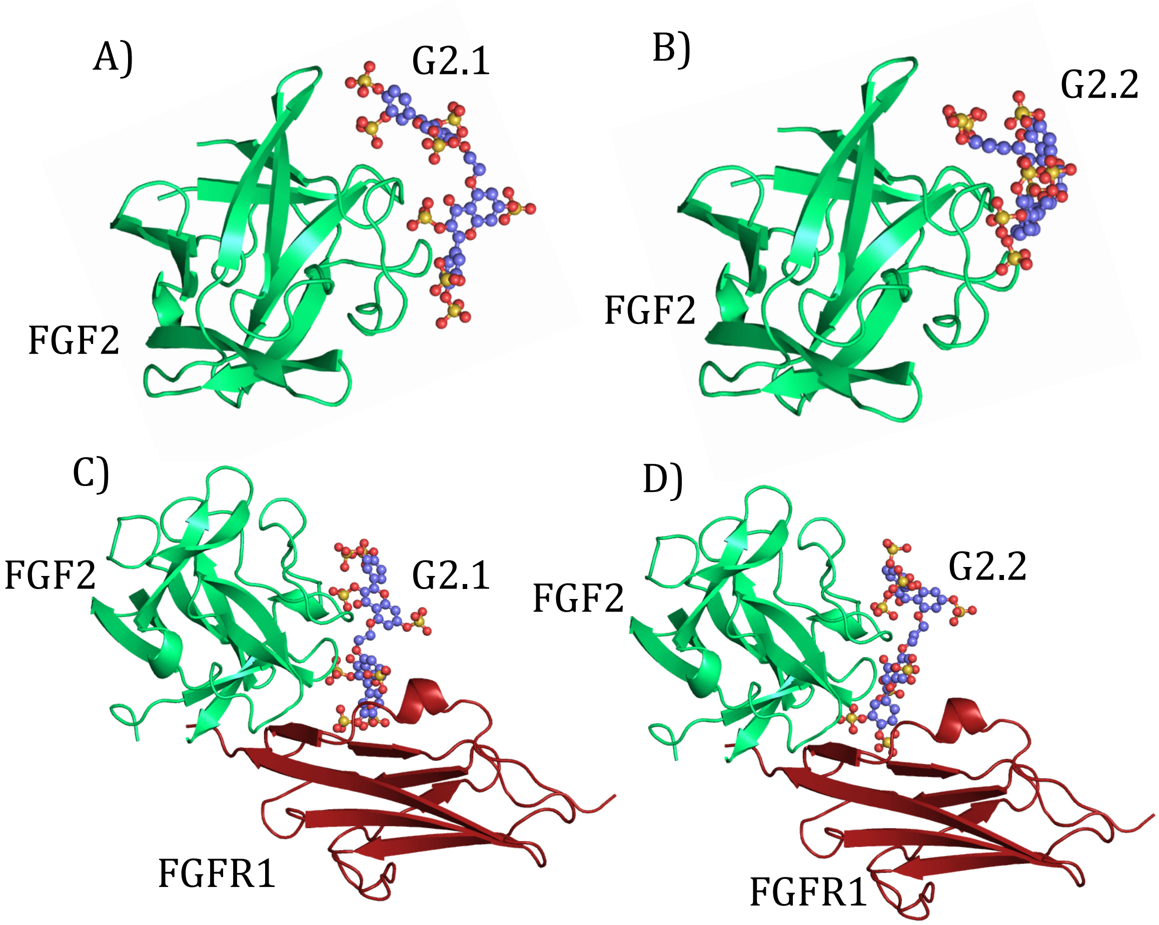

S10 Fig. Molecular docking of NSGMs in the heparin-binding region of FGF2 (1BFC) and FGF2-FGFR1 (1FQ9) complex using GOLD docking tool.
With best score structures shown, A) FGF2-G2.1 docked complex, B) FGF2-G2.2 docked complex, C) FGF1-FGFR1-G2.1 ternary complex and D) FGF2-FGFR1-G2.2 ternary complex. (FGF2, interacting residues were Asn28, Arg121, Lys126, Gly134 and Lys136 for both G2.1 and G2.2. Complex FGF2-FGFR1 key interacting residues showed that G2.1 and G2.2 bound with residues Lys26, Asn27, Arg44, Arg120, Lys125, Lys135 and Ala136 of FGF2 and Glu159, Lys163, His166, Lys172 and Lys175 of FGFR1.

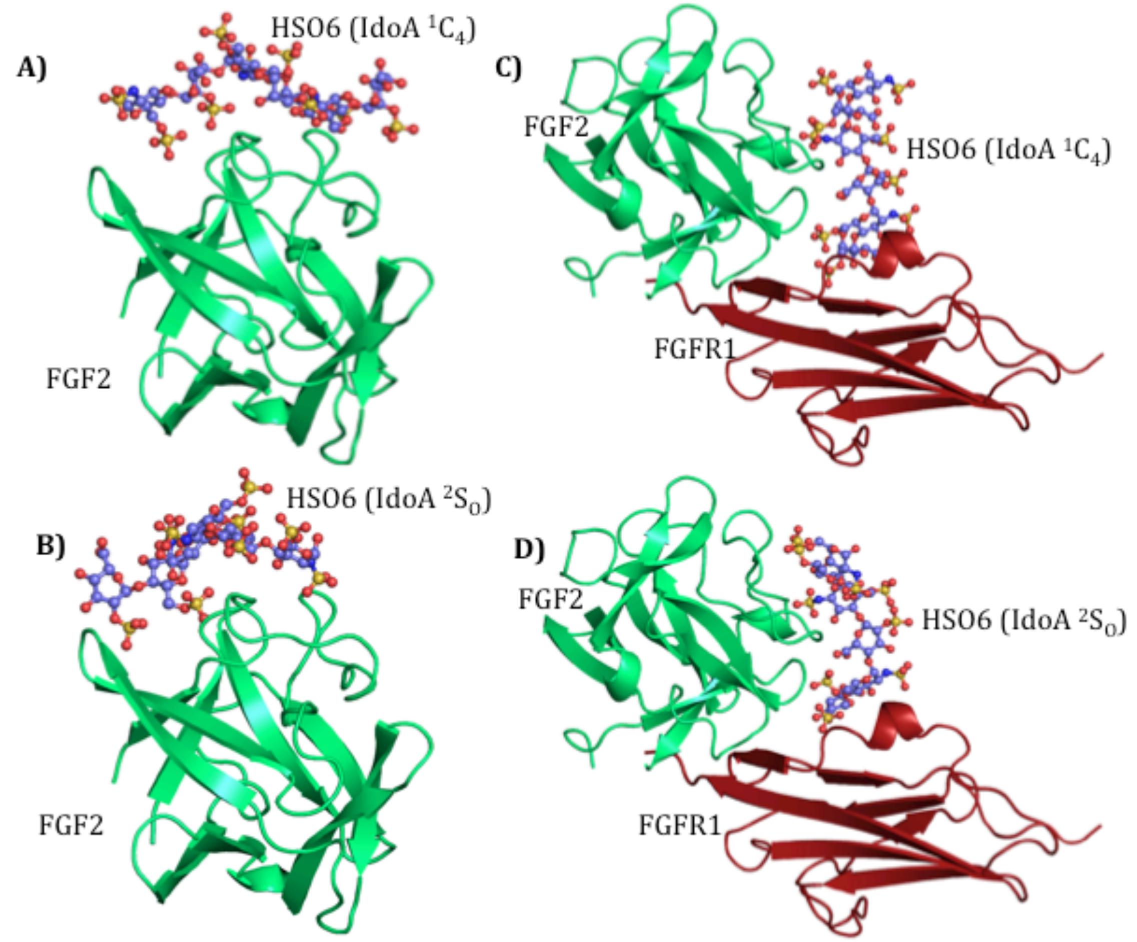

S11 Fig. Molecular docking of HSO6 in the heparin-binding region of FGF2 (1BFC) and FGF2-FGFR1 (1FQ9) complex using GOLD docking tool.
Genetic algorithm-based predicted geometries of HS06 binding to FGF2 (left) and FGF2–FGFR1 complex (right) shown in cartoon. A dual filter GOLD-based docking and scoring strategy, developed earlier in the laboratory (Raghuraman, et al., 2006; Sankaranarayanan and Desai, 2014), was used to study the interaction of HS06 sequence (IdoA2S-GlcNS6S-IdoA2S-GlcNS6S-IdoA2S-GlcNS6S) containing Iduronic acids in either $^1C_4$ or ^2S_O conformations with each of the protein. The geometry of FGF2–FGFR1 complex matched that identified by crystallography (Schlessinger, et al., 2000). HS06 is shown in blue atom-colored sticks. The key interacting residues are shown as white color by atom ball and sticks.

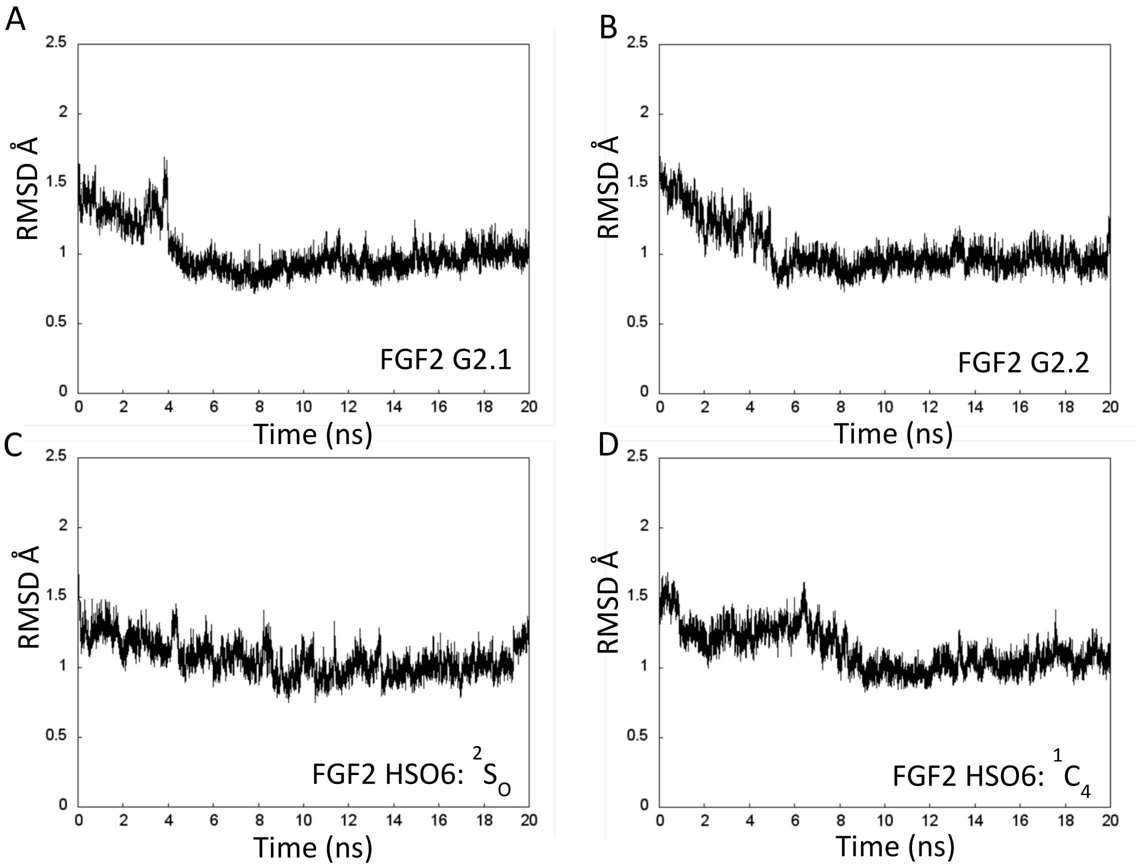

S12 Fig. RMSD of FGF2-NSGMs and FGF2-HSO6 complex.

Conformational fluctuation of FGF2 based on non-hydrogen atoms shown with respect to the average structure. A) G2.1 bound FGF2 B) G2.2 bound FGF2 C) &D) with HSO6 bound FGF2 with IdoA2S in $^{2}S_{O}$-form and IdoA2S in $^{1}C_{4}$-form respectively.

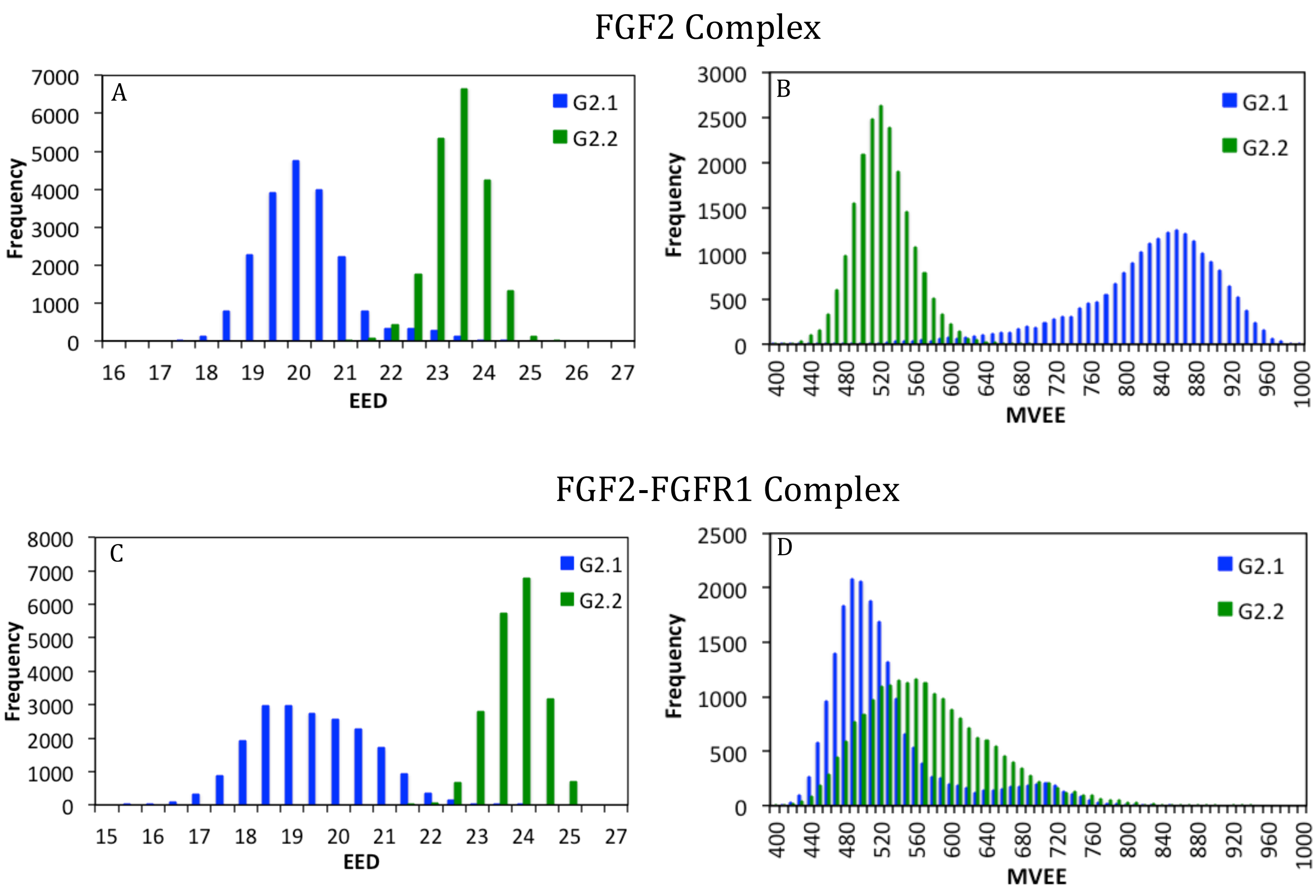

S13 Fig. Distribution of end-to-end distance (EED) and minimum volume enclosing ellipsoid (MVEE) of NSGMs in protein bound form for both FGF2 and FGF2-FGFR1.

A) Frequency distribution of EED (in Å) and B) Frequency distribution of MVEE in cubic units for FGF2 bound NSGMs. C) Frequency distribution of EED (in Å) and D) Frequency distribution of MVEE in cubic units for FGF2-FGFR1 bound NSGMs. The G2.1 and G2.2 are shown in blue and green colors respectively.

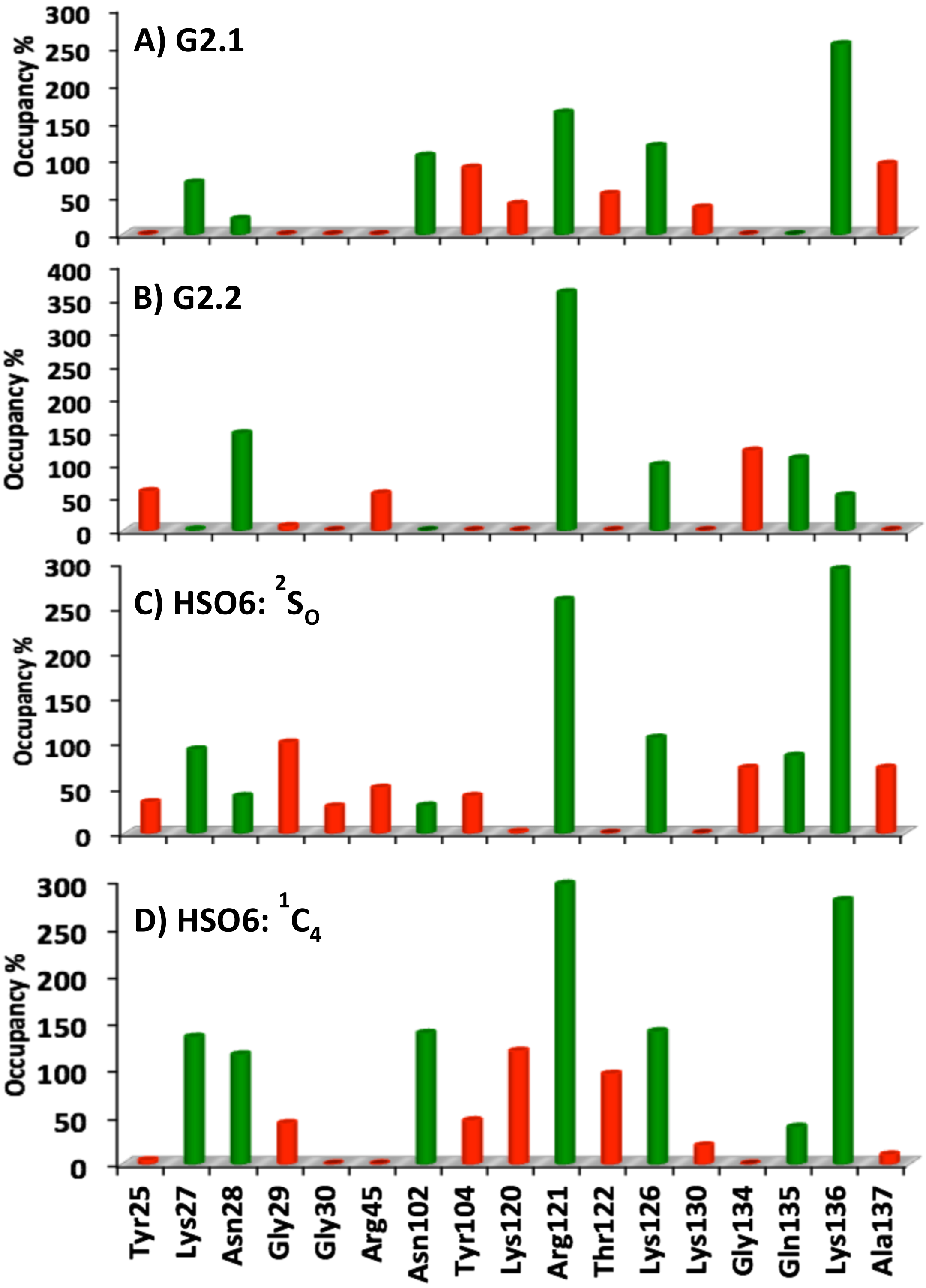

S14 Fig. Intermolecular-hydrogen bond occupancy for the FGF2 complex.

The direct hydrogen bond occupancy of binding region interacting residues of FGF2 towards NSGMs and HSO6 are shown A) G2.1 B) G2.2 C) HSO6: $^{2}S_{O}$ D) HSO6: $^{1}C_{4}$ respectively. The similar identified residues to crystal structure (PDBID 1BFC) are shown in green color and those are not seen are in red color.

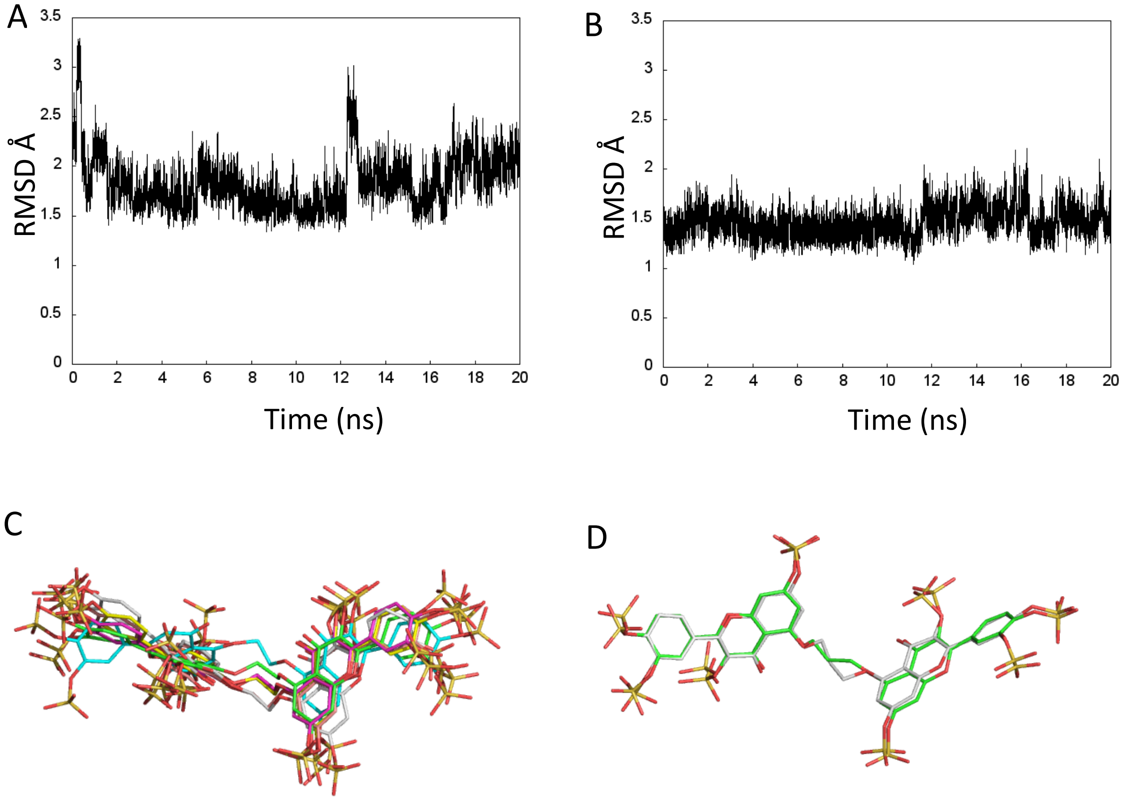

S15 Fig. The conformational flexibility of FGF2 bound dimers.

A) RMSD of non hydrogen atoms of G2.1 with respect to average structure B) RMSD of non hydrogen atoms of G2.2 with respect to average structure C) superposition of G2.1 cluster centroids with initial structure (white) D) superposition of G2.2 cluster centroid with initial structure (white).

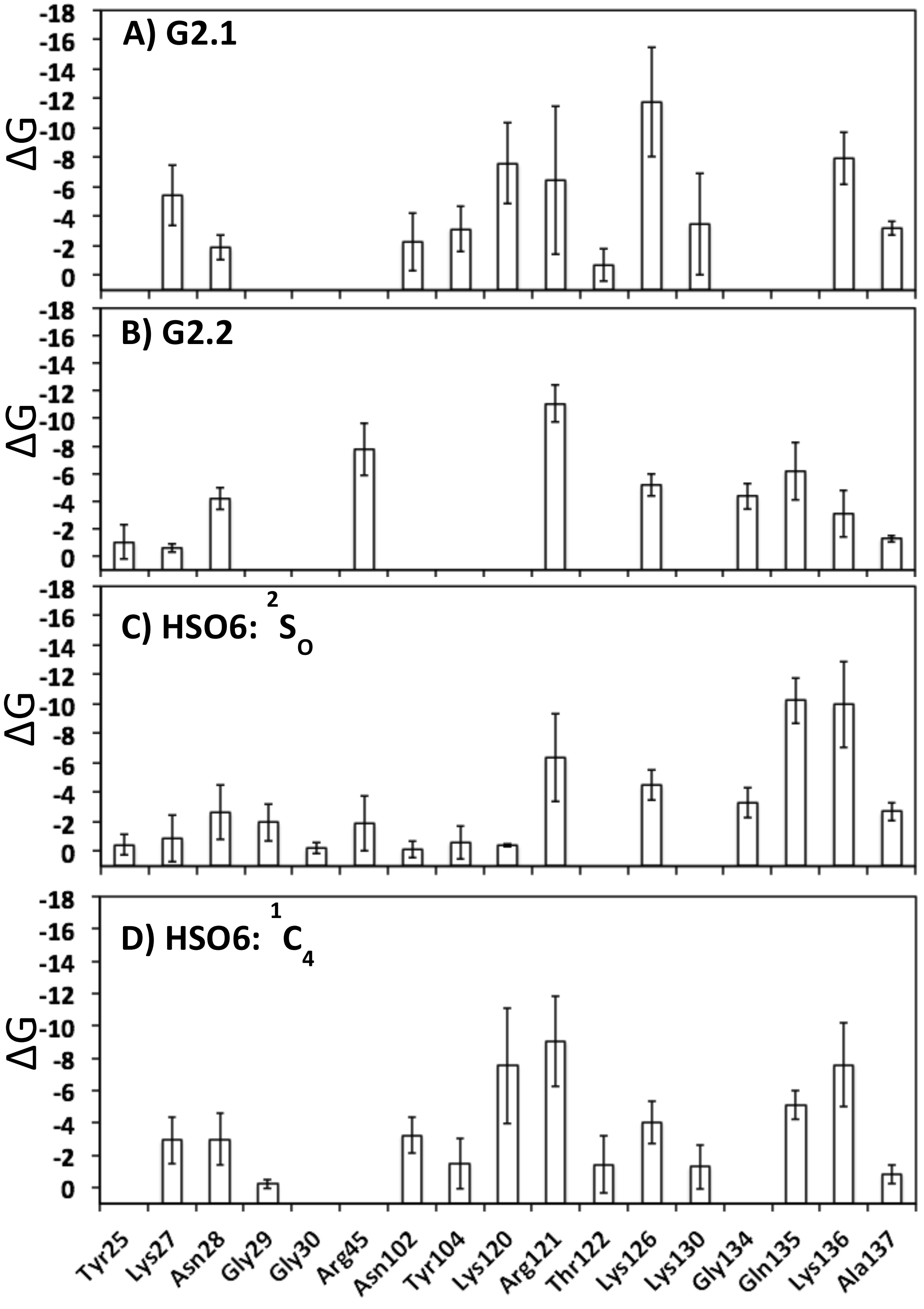

S16 Fig. Single residue energy decomposition of FGF2 complexes.

Shows the impact of individual residues on FGF2 binding. Results are shown for both NSGMs and HSO6 molecules A) G2.1 B) G2.2 C) HSO6: $^{2}S_{O}$ D) HSO6: $^{1}C_{4}$ respectively. (All the values of ΔG are given in kcal/mol, error bar represents the standard deviation).

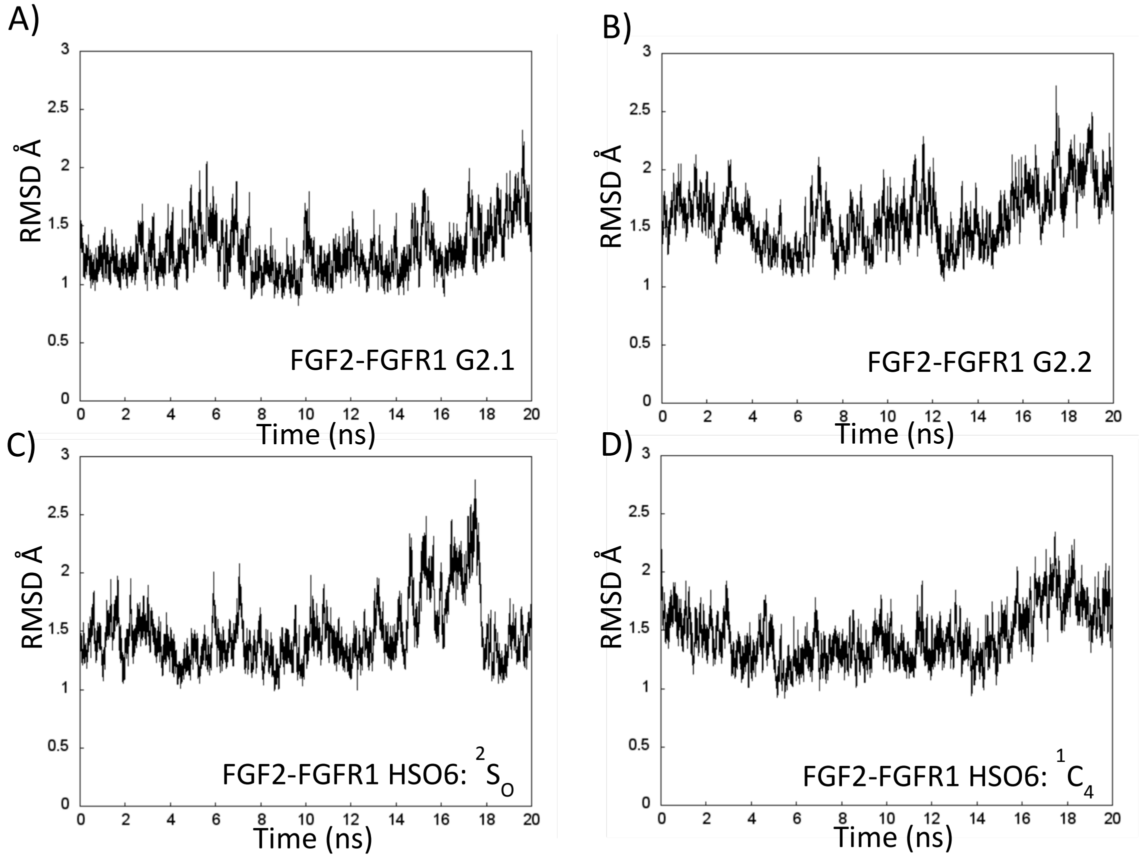

S17 Fig. RMSD of FGF2-FGFR1 NSGMs and FGF2-FGFR1 HSO6 complex.
Conformational fluctuation of FGF2-FGFR1 based on non-hydrogen atoms shown with respect to the average structure. A) G2.1 bound FGF2-FGFR1 B) G2.2 bound FGF2-FGFR1 C) &D) with HSO6 bound FGF2-FGFR1 with IdoA2S in $^{2}S_{O}$-form and IdoA2S in $^{1}C_{4}$-form respectively.

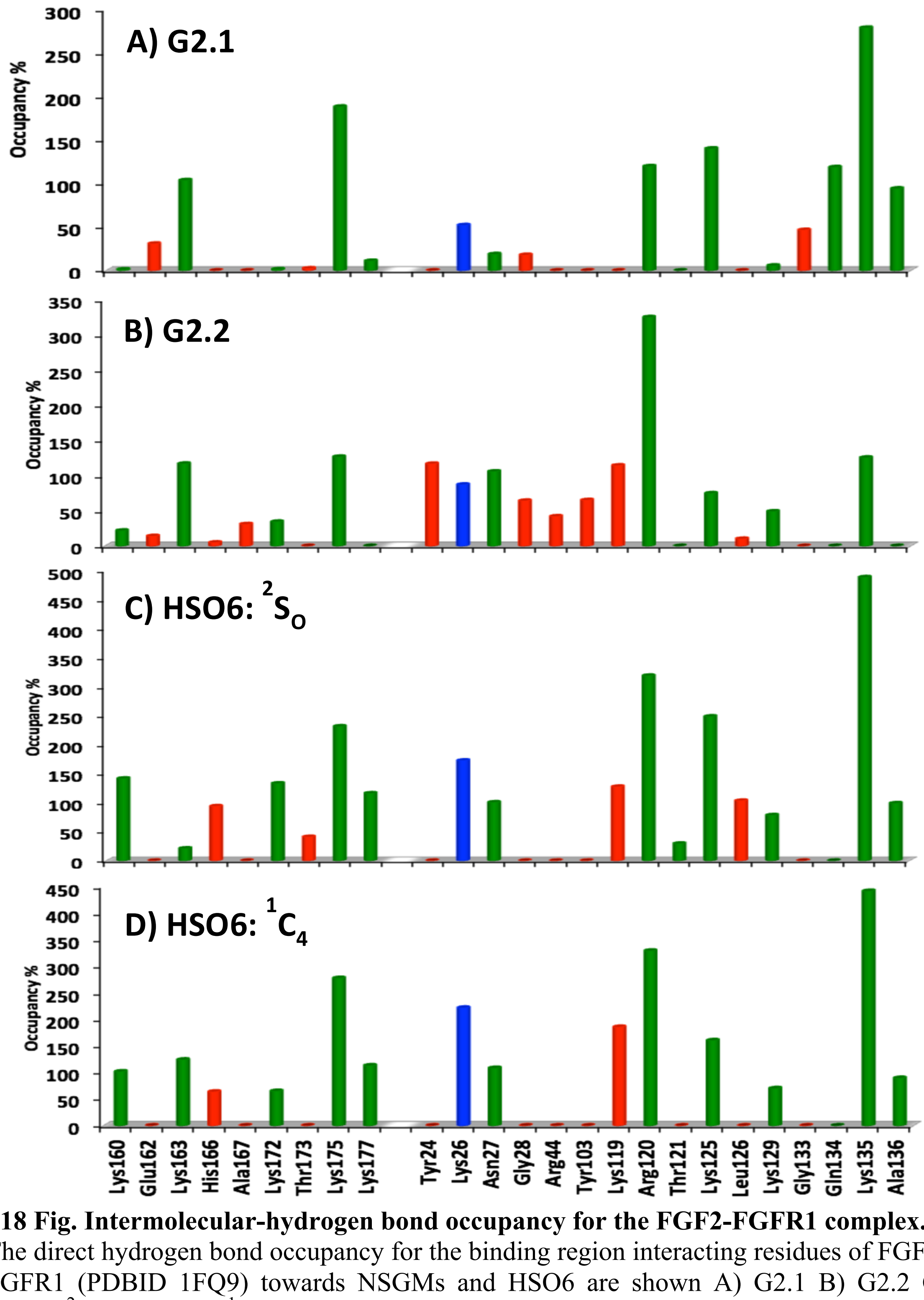

S18 Fig. Intermolecular-hydrogen bond occupancy for the FGF2-FGFR1 complex. The direct hydrogen bond occupancy for the binding region interacting residues of FGF2-FGFR1 (PDBID 1FQ9) towards NSGMs and HSO6 are shown A) G2.1 B) G2.2 C) HSO6: $^{2}S_{O}$ D) HSO6: $^{1}C_{4}$ respectively. The similar identified residues to crystal structure are shown in green color and those are not seen are in red color, Blue color LYS-A-26 represents similar observations from sucrose octasulfate binding (PDBID: 1CVS).

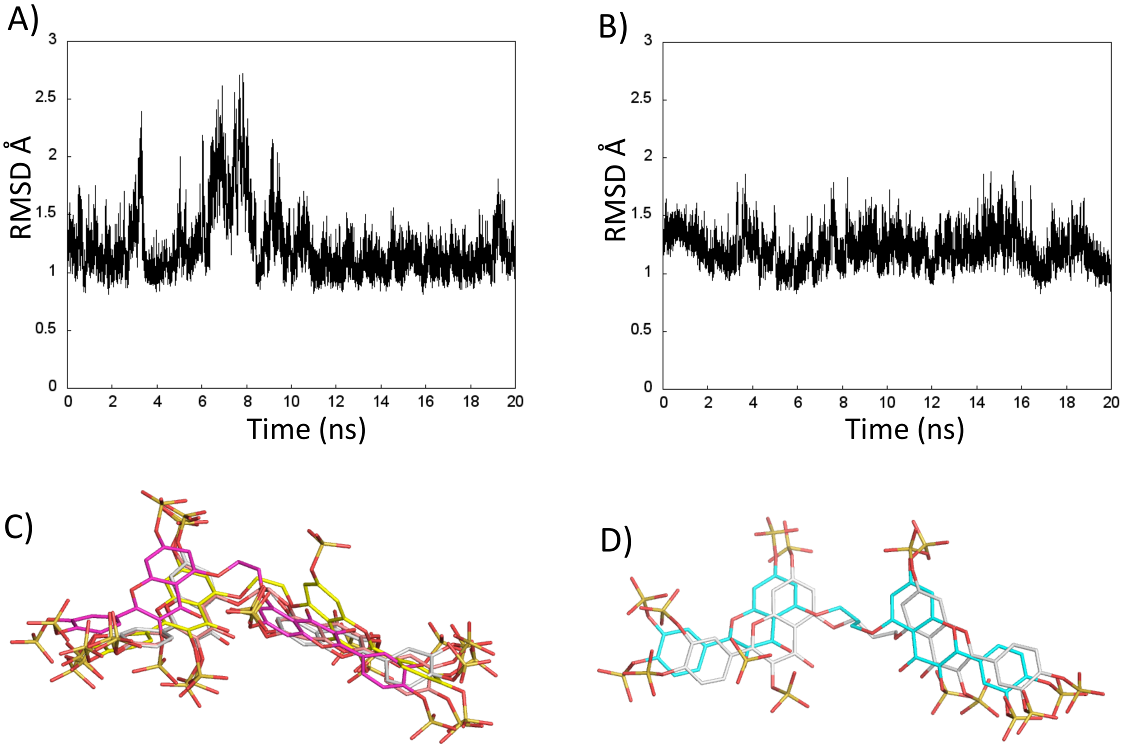

S19 Fig. The conformational flexibility of FGF2-FGFR1 bound NSGMs dimers.
A) RMSD of non hydrogen atoms of G2.1 with respect to average structure B) RMSD of non hydrogen atoms of G2.2 with respect to average structure C) superposition of G2.1 cluster centroids with initial structure (white) D) superposition of G2.2 cluster centroid with initial structure (white).

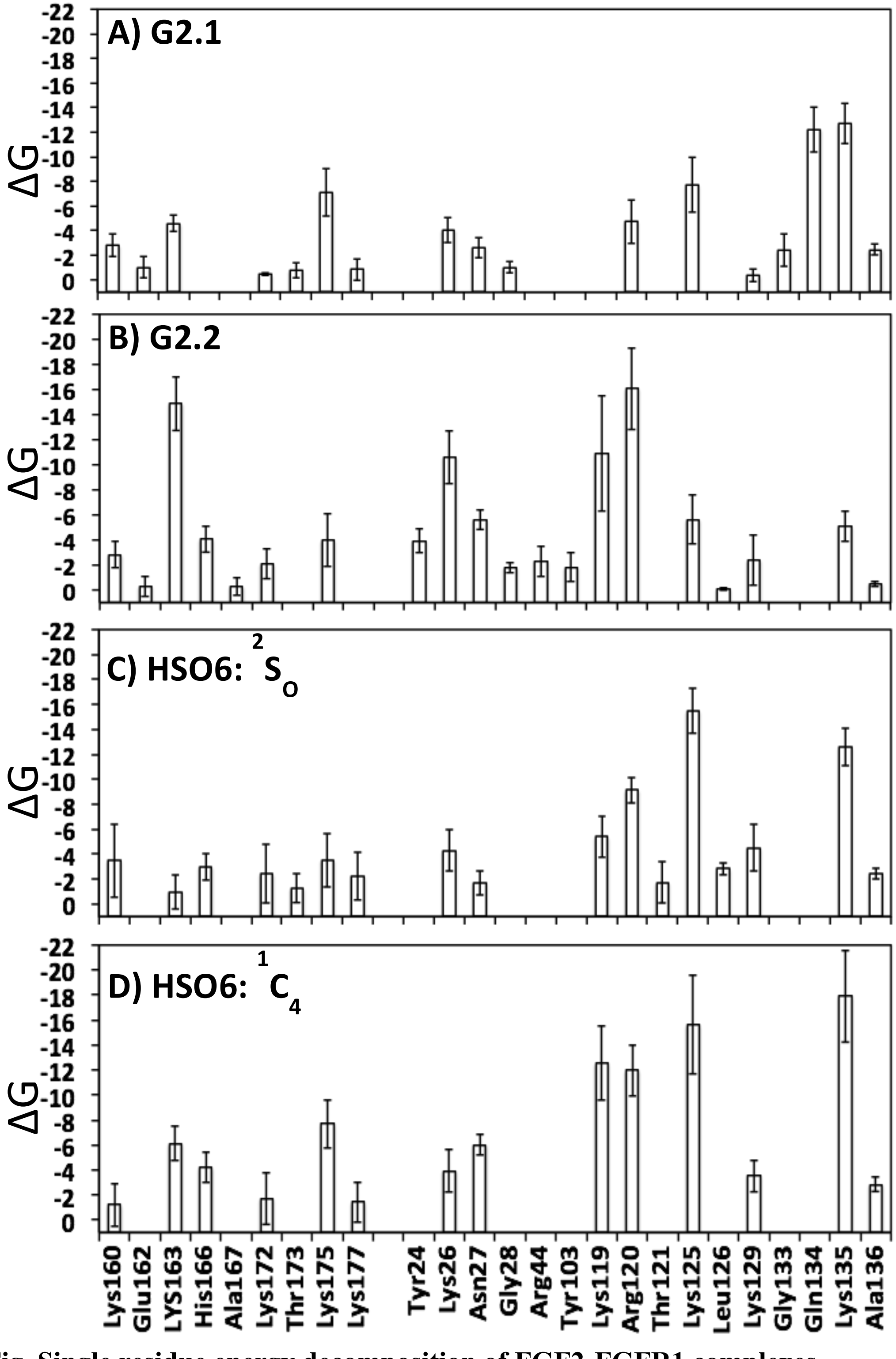

S20 Fig. Single residue energy decomposition of FGF2-FGFR1 complexes.

Shows the impact of individual residues on FGF2-FGFR1 binding. Results are shown for both NSGMs and HSO6 molecules A) G2.1 B) G2.2 C) HSO6: ^2S_O D) HSO6: $^1C_4$ respectively. (All the values of ΔG are given in kcal/mol, error bar represents the standard deviation).
