# Supplementary material for: A molecular dynamics-based algorithm for evaluating the glycosaminoglycan mimicking potential of synthetic, homogenous, sulfated small molecules
Source: PLoS One. 2017 Feb 9;12(2):e0171619. doi: 10.1371/journal.pone.0171619 (PMC5300208; doi:10.1371/journal.pone.0171619)
Supplement: S2 File — (PDF) [file pone.0171619.s002.pdf]

Automated code for measuring minimum volume enclosing ellipsoid (MVEE)

```
fid = fopen('PDBFILE','r');
for i = Number of entries in pdb ( 1 to N )
fid1 = fopen('Tempfile','w'); # To write each PDB's (x,y,z) co-ordinates
for j = 1:M % Number of lines in each PDB entry
fscanf each line of PDBFILE
end
for j = Number of entries in pdb
x = sscanf x-coordinate
fprintf x-coordinate
end
for j = 1 :M
y = sscanf y-coordinate
fprintf y-coordinate
end
for j = 1 :M
z = sscanf z-coordinate
fprintf z-coordinate
end
fclose(fid1);
load fid1;
P = fid1;
[A, C] = MinVolEllipse(P, .01);
MinVolEllipse_plot(A,c)
[U Q V] = svd(A);
r1 = 1/sqrt(Q(1,1));
r2 = 1/sqrt(Q(2,2));
r3 = 1/sqrt(Q(3,3));
% Calculation of volume by multiplying radii
volume = r1*r2*r3; %As a comparative measurements the constants are not
included
%Ploting Minimum Volume Elipsoid
figure
plot3(P(1,:),P(2,:),P(3,:),'*');
hold on
MinVolEllipse_plot(A,C);
Fprintf (r1,r2,r2, volume);
end
fclose(fid2);
```

Note: For more information about the minimum volume enclosing ellipsoid refer: http://www.mathworks.com/matlabcentral/fileexchange/9542-minimum-volume-enclosing-ellipsoid by Nima Moshtagh (2009)
